# Plasma clusterin as a candidate pre-diagnosis marker of colorectal cancer risk in the Florence cohort of the European Prospective Investigation into Cancer and Nutrition: a pilot study

**DOI:** 10.1186/s12885-015-1058-7

**Published:** 2015-02-14

**Authors:** Michela Bertuzzi, Cristina Marelli, Renzo Bagnati, Alessandro Colombi, Roberto Fanelli, Calogero Saieva, Marco Ceroti, Benedetta Bendinelli, Saverio Caini, Luisa Airoldi, Domenico Palli

**Affiliations:** 1 Department of Environmental Health Sciences, IRCCS–Istituto di Ricerche Farmacologiche Mario Negri, Via Giuseppe La Masa 19, 20156 Milano, Italy; 2 Molecular and Nutritional Epidemiology Unit, ISPO - Cancer Research and Prevention Institute, Florence, Italy

**Keywords:** Early plasma biomarkers, Colorectal cancer, Prospective study, Proteomics, Mass spectrometry

## Abstract

**Background:**

Colorectal cancer is one of the major causes of cancer mortality world-wide. Prevention would improve if at-risk subjects could be identified. The aim of this study was to characterise plasma protein biomarkers associated with the risk of colorectal cancer in samples collected prospectively, before the disease diagnosis.

**Methods:**

After an exploratory study on the comprehensive plasma proteome analysis by liquid chromatography-tandem mass spectrometry from ten colorectal cancer cases enrolled at diagnosis, and ten matched controls (Phase 1), a similar preliminary study was performed on prospective plasma samples from ten colorectal cancer cases, enrolled years before disease development, and ten matched controls identified in a nested case–control study within the Florence cohort of the European Prospective Investigation into Cancer and Nutrition (EPIC) study (Phase 2); in Phase 3 the validation of the candidate biomarkers by targeted proteomics on 48 colorectal cancer cases and 48 matched controls from the Florence-EPIC cohort, and the evaluation of the disease risk were performed.

**Results:**

Systems biology tools indicated that both in the Phase 1 and Phase 2 studies circulating protein levels differing in cases more than 1.5 times from controls, were involved in inflammation and/or immune response. Eight proteins including apolipoprotein C-II, complement C4-B, complement component C9, clusterin, alpha-2-HS-glycoprotein, mannan-binding lectin serine-protease, mannose-binding protein C, and N-acetylmuramoyl-L-alanine amidase were selected as promising candidate biomarkers. Targeted proteomics of the selected proteins in the EPIC samples showed significantly higher clusterin levels in cases than controls, but only in men (mean ± SD, 1.98 ± 0.46 and 1.61 ± 0.43 nmol/mL respectively, Mann–Whitney U, two-tailed P = 0.0173). The remaining proteins were unchanged. Using multivariate logistic models a significant positive association emerged for clusterin, with an 80% increase in the colorectal cancer risk with protein’s unit increase, but only in men.

**Conclusions:**

The results show that plasma proteins can be altered years before colorectal cancer detection. The high circulating clusterin in pre-diagnostic samples suggests this biomarker can improve the identification of people at risk of colorectal cancer and might help in designing preventive interventions.

**Electronic supplementary material:**

The online version of this article (doi:10.1186/s12885-015-1058-7) contains supplementary material, which is available to authorized users.

## Background

Colorectal cancer (CRC) is one of the main causes of death from cancer world-wide, with higher incidence and mortality rates in developed countries; it is more frequent in men than women [1].

Most sporadic CRCs develop from a normal epithelium which, after a number of genetic and epigenetic molecular alterations, can turn into adenoma, a benign precursor lesion that can proceed to a malignant tumour [2]. Though no specific CRC etiologic agents have been identified, epidemiological evidence suggests a number of different risk factors, including diet and lifestyle habits, that can be easily modified, so this cancer is potentially preventable [3]. Typically, the progress from adenoma to cancer takes several years, providing a wide time window for preventive intervention.

Diet and lifestyle changes may be effective for primary prevention and screening programs have reduced cancer mortality, but CRC continues to account for more than 9% of all new cancers [1,3]. Preventing CRC therefore requires the identification of suitable biomarkers that must be non-invasive, highly sensitive and specific. The biomarkers currently in use, for instance faecal haemoglobin, and serum tumour markers (CEA and CA 19.9) do not fulfil these requirements, since they are not sufficiently reliable for early detection of CRC and lack specificity and sensitivity [4].

Mass spectrometry-based proteomics offers a means of discovering robust disease biomarkers and this approach is increasingly used in cancer research. Several CRC proteomic studies have analysed samples from experimental models or from human surgical specimens to identify differences in the protein profile induced by cancer [5] and references herein. However, in clinical practice, biomarkers should be easy to measure and this can be achieved mainly by using blood, urine, and faeces [5].

So far, serum or plasma protein biomarkers have been sought mostly in CRC case–control studies, using samples collected at the diagnosis, when the tumour was already developed, but this limits the predictive value of the biomarker [6-9]. By contrast, the prospective study design, which involves people free of disease, could identify biomarkers predictive of disease development.

We used a mass spectrometry-based proteomic approach to identify early biomarkers of CRC in human plasma, dividing the investigation into three phases: first, in an exploratory study with a case–control design we compared the comprehensive plasma proteome from ten CRC cases enrolled at diagnosis, and ten age- and sex-matched controls, and identified differential circulating proteins (disease biomarkers) by liquid chromatography-electrospray ionization-tandem mass spectrometry (LC-ESI-MS/MS); second, we did a similar preliminary study with a nested case–control design to identify candidate predictive biomarkers in plasma from ten CRC cases, enrolled years before the disease developed, and ten age- and sex-matched controls identified in the frame of a nested case–control study on CRC carried out in the Florence cohort of the European Prospective Investigation into Cancer and Nutrition (EPIC) study; in the third phase, we validated the identified candidate biomarkers by liquid chromatography-selected reaction monitoring-mass spectrometry (LC-SRM-MS) on each individual sample of a series of 48 CRC cases and 48 matched controls from the Florence-EPIC cohort, and used these data to estimate the disease risk.

## Methods

### Study populations

*Phase 1: Exploratory study.* In this phase we examined ten newly diagnosed CRC cases and ten age- and sex-matched controls, identified in a hospital-based case–control study on CRC ongoing in the metropolitan area of Florence in the period 2006–2009. All cases were recruited when admitted to the Surgery Departments of the main hospitals in the area. All cases had histologically confirmed adenocarcinoma of the colon-rectum. The controls were randomly selected from a series of healthy adults residing in the study area. The controls were matched to CRC cases by sex and age. The demographic characteristics of Phase 1 subjects are shown in Table 1. The study was approved by the Local Ethical Committee, Area Vasta Centro Regione Toscana. All participants provided a signed informed consent form to use their blood samples and individual data for scientific purposes.

**Table 1:** **Demographic characteristics of the study subjects (CRC cases and controls) by phase**

*Phase 1*				
Characteristic	Cases N	Controls N	Total N	P-value^a^
**Sex**				
M	7	9	16	0.58
F	3	1	4	
**Smoker**				0.20
Current	2	1	3	
Former	5	2	7	
Never	3	7	10	
**Total**	10	10	20	
**Age** (yrs.) mean (SD)	61.6 (11.1)	60.2 (10.9)	60.9 (10.8)	0.89
***Phase 2***				
**Characteristic**	**Cases N**	**Controls N**	**Total N**	**P-value** ^**a**^
**Sex**				1.0
M	4	4	8	
F	6	6	12	
**Smoker**				0.08
Current	4	0	4	
Former	1	2	3	
Never	5	8	13	
**Total**	10	10	20	
**Age** (yrs.) mean (SD)	53.3 (7.8)	53.3 (7.6)	53.3 (7.5)	0.91
***Phase 3***				
**Characteristic**	**Cases N** ^**c**^	**Controls N**	**Total N**	**P-value** ^**a**^
**Sex**				1.00
M	20	20	40	
F	28	28	56	
**Smoker**				0.22
Current	16	10	26	
Former	15	13	28	
Never	17	25	42	
**Waistline** ^**b**^				0.67
≤ OMS cut-off	35	39	74	
> OMS cut-off	8	7	15	
**BMI** ^**b**^				0.13
Normal	17	24	41	
Overweight	26	17	43	
Obesity	3	6	9	
**School**				0.12
Primary	16	7	23	
Secondary	23	32	55	
High	9	9	18	
**Total**	48	48	96	
**Age** (yrs.) mean (SD)	55.1 (6.2)	55.2 (6.2)	55.1 (6.1)	0.98
**Daily Consumption (g)**				
**Characteristic**	**Cases**	**Controls**	**Total**	**P-value** ^**a**^
Fruit intake	258.3 (114.8)	380.4 (192.6)	319.4 (153.7)	0.0003
Vegetables	160.9 (75.3)	232.4 (117.6)	196.7 (96.5)	0.0006
Red meat	74.3 (49.4)	67.1 (45.0)	70.7 (47.2)	0.46
Alcohol	22.8 (22.3)	15.2 (16.5)	19.0 (19.4)	0.06

^a^P-values from chi-square or Mann–Whitney test, as appropriate.

^b^Some data are missing.

^c^CRC location according to ICD-O classification: Cecum, n = 4; Ascending colon, n = 6; Hepatic flexure colon, n = 1; Transverse colon, n = 0; Splenic flexure colon, n = 1; Descending colon, n = 4; Sigmoid colon, n = 15; Colon NOS, n = 5; Rectosigmoid junction, n = 5; Rectum, n = 7.

*Phase 2 and Phase 3: Nested case–control study in EPIC-Florence.* The rationale and methods of the EPIC study have been described elsewhere [10]. Briefly, EPIC is a multicentre prospective cohort study carried out in 23 centres across ten European countries and coordinated by the International Agency for Research on Cancer (IARC, Lyon, France), aimed at investigating the relation between diet, lifestyle and environmental factors, and the incidence of different cancers. EPIC-Florence is one of the five Italian centres [11]. In the period 1993–1998, EPIC-Florence completed the recruitment of 13,597 volunteers aged 35–65 years. Detailed information was recorded for each individual volunteer about diet and life-style habits, anthropometric measurements and a blood sample was collected. Standardized procedures were used to identify newly diagnosed cases of cancer at all sites, including colon-rectum, in the follow-up of the cohort.

Table 1 shows the demographic characteristics of the Phase 2 and 3 subjects. They were participants of EPIC-Florence study, being from the Florence metropolitan area. The study was approved by the local Florence Ethical Committee (Azienda U.S.L. 10 Firenze). All participants provided a signed informed consent form to use their blood samples and individual data for scientific purposes. The 48 CRC cases of the present study (and their matched controls) were randomly selected from a series of case-sets identified in a nested case–control study on CRC carried out in EPIC [12]. Controls had originally been selected by incidence density sampling from all cohort members alive and free of cancer at the time of diagnosis of the cases and were matched by age, sex, time of day at blood collection, and fasting status at the time of blood collection. Women were matched by menopausal status.

### Proteomic analysis

**Figure 1:**
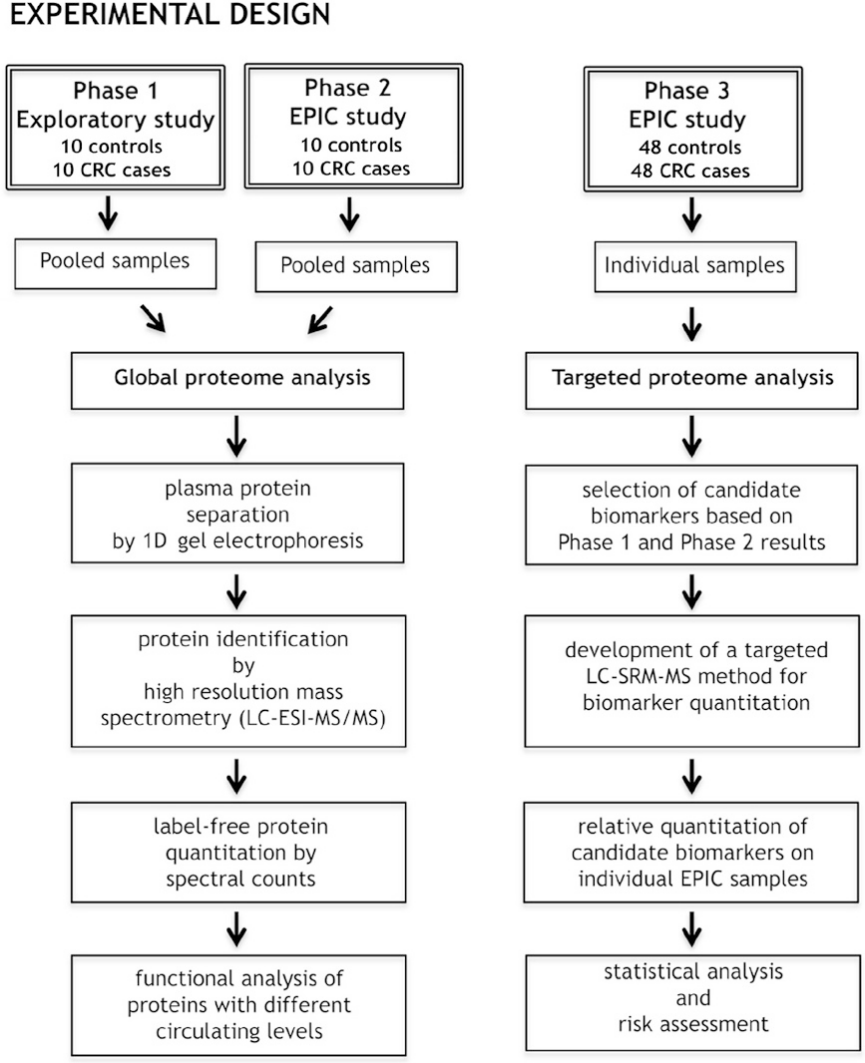
**Flow diagram of the experimental design.**

Sample preparation, protein separation, identification of proteins with different circulating levels by global proteome analysis, and relative quantitation of candidate biomarkers by targeted proteomics, are fully described in Supplementary Methods (Additional file 1). A summary flow diagram of the experimental section is shown in Figure 1.

### Functional and Pathway analysis

MetaCore version 6.12 (GeneGo, St Joseph, MI, USA) was used to map the differentially expressed proteins into biological networks and for functional interpretation of the protein data. Functional and Pathway analyses are described in Supplementary Methods (Additional file 1).

### Statistical analysis

*Phase 1 and Phase 2.* Changes in circulating levels of proteins, separated by one-dimensional gel electrophoresis (1DE) were based on the average normalised spectral counts (3 replicate runs) of the proteins identified by LC-ESI-MS/MS. Proteins showing at least a 1.5-fold up or down change (FC, fold change, ratio of the averaged spectral counts in CRC samples to the averaged spectral counts in control samples) were considered to have different levels.

Partial Least Squares-Discriminating Analysis (PLS-DA) was applied to Phase 1 and Phase 2 protein spectral counts, to find proteins discriminating CRC cases form controls. We used Simca-P v13 (MKS Umetrics AB, Sweden) for data analysis after Pareto normalization.

*Phase 3.* Between-groups comparisons of the selected protein relative amounts obtained after LC-SRM-MS were computed on the mean of three analytical replicates using the non-parametric Mann–Whitney U test, two-tailed; biomarker validation was done by Receiver Operating Characteristic (ROC) curve analysis. We used the Prism software v6 (GraphPad Software Inc. La Jolla, CA, USA), setting the significance at P <0.05.

The association between each protein and cancer status was evaluated in the whole series by separate multivariate logistic models stratified by case-set, i.e. pair of cases and controls matched for sex and age, and adjusted for potential confounders. Each protein was used as continuous or dichotomous (above/below the median value) variable. We ran four different logistic models, the first three using each protein as continuous variable, and the last using each protein as dichotomous variable. The logistic models were adjusted by smoking, waistline and education (model 1); by smoking, BMI, and education (model 2); by smoking, waistline, education and daily intake of fruit, vegetables, red meat, and alcohol (model 3); by smoking, waistline, and education (model 4). Smoking status was included as dummy variable (current-, former- *vs.* never-smoker), BMI as dummy variable (obesity, overweight *vs.* normal), waistline as dichotomous variable according to the WHO cut-off (88 cm for women, 102 cm for men), education as dummy variable (high school, secondary school *vs.* primary school), and daily intake of fruit, vegetables, red meat, and alcohol as continuous variables (g/day). We also applied multivariate logistic models separately for men and women; in these analyses the models were also adjusted by age.

All logistic analyses were done using SAS (SAS/STAT version 9.1) statistical program. A P-value <0.05 was considered significant.

## Results

### Phase 1. Exploratory study: global proteome analysis by 1DE/LC-ESI-MS/MS

Mass spectrometric analysis after 1DE separation led to the identification of 138 proteins common to CRC cases and controls. Of these, only 94, listed in Additional file 2: Table S1 together with their relative quantitation by spectral count, met the restriction criteria reported in Supplementary Methods (Additional file 1). Plasma levels of 13 proteins, based on spectral counts, were higher in CRC patients than controls (FC ≥1.5). Eight proteins had lower levels in cases than controls, with FC ≤ −1.5 (Additional file 2: Table S1).

The quantitative data trend was explored by PLS-DA analysis on protein spectral counts. The score scatter plot showed good separation of CRC cases and controls with cumulative statistical parameters R^2^(X) = 0.699; R^2^(Y) = 0.996; Q^2^ = 0.922 (Additional file 3: Figure S1). The PLS-DA Variable Importance in the Projection (VIP) values are listed in the Additional file 2: Table S1. Proteins with VIP > 1 significantly contributed to the separation of the two groups.

### Phase 2. EPIC-Florence study: global proteome analysis by 1DE/LC-ESI-MS/MS

Protein separation of the two plasma pools by 1DE followed by LC-ESI-MS/MS analysis identified 178 proteins, 104 meeting the restriction criteria reported in Supplementary Methods (Additional file 1). The identified proteins and their relative quantitation by spectral counts are listed in Additional file 2: Table S2. Twelve proteins had FC ≥1.5 and four proteins had FC ≤ −1.5.

As shown in Additional file 3: Figure S2, the PLS-DA score scatter plot of the 104 identified proteins showed significant separation of EPIC-CRC cases and controls with cumulative R^2^(X) = 0.604; R^2^(Y) = 0.985; Q^2^ = 0.685. A number of proteins showed a good discriminatory ability between the two groups. These proteins had VIP values >1 (Additional file 2: Table S2) and were considered significant.

**Figure 2:**
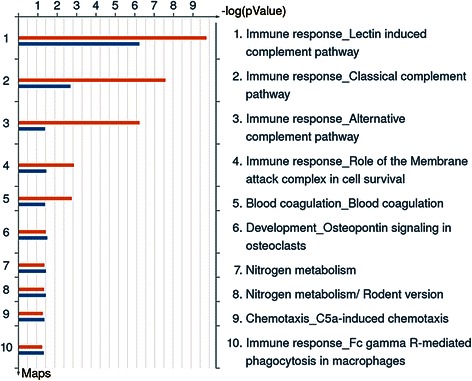
**MetaCore “Enrichment analysis” on proteins with altered plasma levels (FC ≥ 1.5 or ≤ −1.5).** The histograms represent the most significant biological process maps in which the proteins are involved. The results are ranked by the -log(p-value). Red histograms, Phase 1 Exploratory study; blue histograms, Phase 2 EPIC study.

Comparison of the two global proteome studies indicated that 83 out of 114 total proteins were common to the Phase 1 exploratory and Phase 2 EPIC studies, 20 were present only in EPIC samples, and 11 were identified only in the exploratory study. MetaCore Enrichment Analysis only on proteins with FC ≥1.5 (31 proteins whose levels were higher or lower in cases than in controls) showed that most of them were involved in the complement systems (classical, lectin, and alternative complement systems). Figure 2 shows the ten top most significant biological process maps. The enrichment network of Additional file 3: Figure S3, using the protein lists from exploratory and EPIC studies, indicated that nine proteins were brought together into the Complement system network.

### Phase 3. Relative quantitation of candidate biomarkers by LC-SRM-MS

The global plasma proteome data of the Phase 2 EPIC samples showed only a few changes in circulating protein levels, so these results alone did not allow the selection of candidate biomarkers. However, our data as a whole suggested there were proteins deserving further analysis. So we took account of all the possible suggestions given by Phase 2 data. We considered at least one of the following inclusion criteria: (i) proteins with normalised spectral count coefficient of variance <35% and FC ≥1.5 or ≤ −1.5; (ii) proteins giving VIP > 1 after PLS-DA. More stringent criteria were not applied, so as to have a more inclusive list of candidate biomarkers. After preliminary LC-SRM-MS analyses (not shown), proteins giving unreliable results were discarded. We ended up with the eight proteins listed in Table 2 together with the amino acid sequence of the peptides selected for quantitation, their molecular weight, precursor and product ion mass/charge ratio, and collision energy.

**Table 2:** **Candidate biomarkers selected for LC-SRM-MS analysis**

Protein name	UniProt Entry name	FC^a^	VIP^b^	Protein function^c^	Proteotypic peptide^d^	Peptide molecular weight	Transitions^f^		CE (V)^h^
							Precursor ion	Product ion	
							m/z^g^	m/z^g^	
Apoliprotein C-II	APOC2	2.44	0.94	Lipid transport	TYLPAVDEK	1034.5	518.3	771.4	25
							**518.3**	**658.34**	**25**
Clusterin	CLU	1.32	1.63	Complement pathway, innate immunity	TLLSNLEEAK	1118.8	559.4	790.4	20
							**559.4**	**903.5**	**20**
Complement C4-B	CO4-B	1.08	1.57	Complement pathway, innate immunity	VGDTLNLNLR	1113.8	557.9	629.4	15
							**557.9**	**742.5**	**15**
Complement Component C9	CO9	1.31	1.65	Complement activation, classical pathway	VVEESELAR	1030.5	516.27	704.35	25
							**516.27**	**833.4**	**25**
Alpha-2-HS-glycoprotein (Fetuin A)	FETUA	−1.14	1.46	Acute-phase response	HTLNQIDEDK	1196.6	598.9	845.4	20
							**598.9**	**958.2**	**20**
Mannan-binding lectin serine-protease	MASP2	1.72	0.50	Lectin complement pathway, innate immunity	AGYVLHR^e^	814.4	408.23	425.8	15
							**408.23**	**312.9**	**15**
Mannose-binding protein C	MBL2	3.30	0.62	Lectin complement pathway, innate immunity	SPDGDSSLAASER	1290.8	646.9	533.3	25
							**646.9**	**733.38**	**25**
N-acetylmuramoyl-L-alanine amidase	PGRP2	1.62	1.03	Petidoglycan digestion, innate immunity	TFTLLDPK	933.5	466.67	686.4	25
							**466.67**	**585.4**	**20**
Bovine Fetuin	FETUA-B	Internal Standard			TPIVGQPSIPGGPVR	1474.8	737.9	582.3	25
							**737.9**	**879.5**	**25**

^**a**^FC, fold change of protein plasma level in the global proteome study of the EPIC population.

^**b**^VIP, variable importance in the projection, PLS-DA analysis (global proteome study of the EPIC population).

^**c**^Deduced from UniProt database.

^d^Amino acid sequence of the peptide selected for quantitation by LC-SRM-MS.

^e^Although this peptide has only seven amino acid residues, it was selected for SRM analysis because it gave the best response.

^f^The transition used for quantitation is shown in **bold** type; the other transition was used to maximise the specificity of the method.

^g^m/z, mass to charge ratio of the selected peptide.

^h^CE, collision energy.

Additional file 3: Figure S4 illustrates typical SRM transition traces showing the separation of the eight selected peptides plus the internal standard peptide and starting/ending points of the time segments (see Supplementary Methods, Additional file 1). The LC-SRM-MS method was suitable for the relative quantitation of the proteins, as shown by the linear response obtained with increasing amounts of plasma (R between 0.88 and 0.99, Additional file 3: Figure S5.

**Figure 3:**
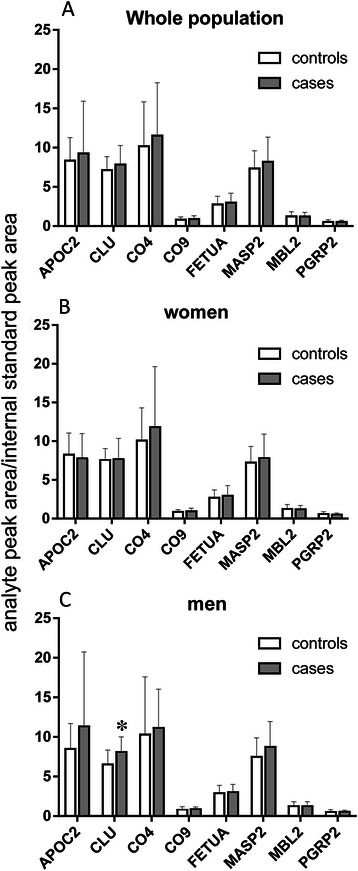
**Bar chart showing the relative amounts of proteins analysed by targeted proteomics (LC-SRM-MS) in the whole EPIC population (Panel A), in women only (Panel B) and in men only (Panel C).** Bars and error bars refer to mean ± SD of the ratio of the analyte peak area to that of the internal standard. The asterisk indicates a significant difference between EPIC CRC male cases and controls (P = 0.0167 Mann–Whitney U, two-tailed).

Bars in Figure 3, panel A show the relative amounts of the selected proteins in the whole EPIC-Florence cohort. There was no significant difference between CRC cases and controls though clusterin (CLU) reached a borderline significance (Mann–Whitney U, two-tailed P = 0.057). When the comparison was done separately on women and men, no difference was seen in women (Figure 3, panel B), but a significant difference emerged in men for CLU (Figure 3, panel C, Mann–Whitney U, two-tailed P = 0.0167).

As shown in Additional file 2: Table S3 the results did not change when the 20 samples from Phase 2 were not included in the statistical analyses, suggesting that their inclusion did not bias the results.

To establish whether the CLU levels found in this study were in agreement with previously reported data, we developed a method for absolute quantitative analysis. The method showed a linear response between 0.2 and 3.2 pmol CLU/sample (R = 0.999). The absolute plasma CLU concentration in the EPIC samples was 1.83 ± 0.5 nmol/mL. Plasma CLU was respectively 1.92 ± 0.57 and 1.75 ± 0.40 nmol/mL in CRC cases and controls (Mann–Whitney U, two-tailed P = 0.057). In the males, EPIC CRC cases had significantly higher CLU than controls (1.98 ± 0.46 and 1.61 ± 0.43 nmol/mL respectively, Mann–Whitney U, two-tailed P = 0.0173). No difference was seen in women (1.88 ± 0.64 and 1.85 ± 0.36 nmol/mL respectively in CRC cases and controls).

### Validation of candidate biomarkers analysed in phase 3

Tables 3, 4, and 5 report the P-values from separate multivariate logistic models for each protein considered as continuous (models 1–3) or dichotomous variable (above/below the median value, model 4) in the whole series (96 samples) in women (56 samples) and in men (40 samples), respectively.

**Table 3:** **Logistic regression models in the whole EPIC series** ^**a**^ **: P-values**

Model^b^	APOC2	CLU	CO4-B	CO9	FETUA	MASP2	MBL2	PGRP2
1	0.62	0.33	0.56	0.41	0.41	0.42	0.75	0.93
2	0.26	0.17	0.29	0.07	0.35	0.18	0.58	0.59
3	0.32	0.89	0.32	0.17	0.84	0.37	0.87	0.69
4	0.59	0.68	0.27	0.92	0.42	0.57	0.81	0.84

^**a**^96 samples, 40 men + 56 women.

^**b**^Model 1 (each protein considered as continuous): stratified by case-set, adjusted by smoking, waistline, education.

Model 2 (each protein considered as continuous): stratified by case-set, adjusted by smoking, BMI, education.

Model 3 (each protein considered as continuous): stratified by case-set, adjusted by smoking, waistline, education, daily intake of fruit, vegetables, red meat, and alcohol.

Model 4 (each protein considered as dichotomised above/below the median value): stratified by case-set, adjusted by smoking, waistline, education.

**Table 4:** **Logistic regression models in EPIC women** ^**a**^ **: P-values**

Model^b^	APOC2	CLU	CO4-B	CO9	FETUA	MASP2	MBL2	PGRP2
1	0.22	0.65	0.59	0.44	0.27	0.53	0.84	0.37
2	0.46	0.76	0.41	0.21	0.18	0.40	0.85	0.48
3	0.92	0.54	0.51	0.10	0.35	0.44	0.81	0.22
4	0.32	0.91	0.66	0.78	0.52	0.67	0.80	0.61

^**a**^56 samples.

^**b**^Model 1 (each protein considered as continuous): adjusted by age, smoking, waistline, education.

Model 2 (each protein considered as continuous): adjusted by age, smoking, BMI, education.

Model 3 (each protein considered as continuous): adjusted by age, smoking, waistline, education, daily intake of fruit, vegetables, red meat, and alcohol.

Model 4 (each protein considered as dichotomised above/below the median value): adjusted by age, smoking, waistline, education.

**Table 5:** **Logistic regression models in EPIC men** ^**a**^ **: P-values**

Model^b^	APOC2	CLU	CO4-B	CO9	FETUA	MASP2	MBL2	PGRP2
1	0.31	**0.02**	0.62	0.96	0.52	0.20	0.47	0.25
2	0.26	**0.01**	0.62	0.51	0.78	0.18	0.87	0.20
3	0.80	0.19	0.13	0.29	0.14	0.08	0.51	0.17
4	0.64	0.089	0.089	0.86	0.25	0.21	0.99	0.27

P-values <0.05 are shown in bold type.

^a^40 samples.

^b^Model 1 (each protein considered as continuous): adjusted by age, smoking, waistline, education.

Model 2 (each protein considered as continuous): adjusted by age, smoking, BMI, education.

Model 3 (each protein considered as continuous): adjusted by age, smoking, waistline, education, daily intake of fruit, vegetables, red meat, and alcohol.

Model 4 (each protein considered as dichotomised above/below the median value): adjusted by age, smoking, waistline, education.

No significant association emerged for the whole series or the females (Tables 3 and 4 respectively). In men, however (Table 5), there was a significant positive association for CLU using models 1 and 2, with an 80% increase in the risk of CRC with protein’s unit increase (OR: 1.83; 95% CI: 1.12-3.00, and OR: 1.80; 95% CI: 1.14-2.85, respectively). The interval between sample collection and disease diagnosis (mean time before CRC diagnosis 3.0 years, SD: 2.0 years; range 0.3-8.2 years) did not affect CLU levels in the whole case series (P = 0.82), or after stratification by sex (men P = 0.30; women P = 0.53).

**Figure 4:**
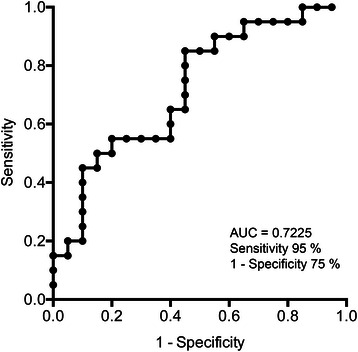
**Clusterin ROC curve in men (AUC = 0.7225; 95% CI: 0.56-0.88; P = 0.0161).**

We further validated CLU as a very early biomarker to distinguish CRC cases from controls by ROC analysis. The results showed a significant AUC of 0.7225 (95% CI: 0.56-0.88; P = 0.0161) only in men. The most convenient cut-off generated a sensitivity of 95% and a specificity of 75%. The ROC curve is shown in Figure 4. Individual ROC curves of the remaining candidate biomarkers showed AUC slightly >0.5. Various AUC combinations (CLU plus the other candidate biomarkers) did not improve sensitivity and specificity. Additional file 2: Table S4 reports candidate biomarker combinations with significant AUC.

## Discussion

Global proteomics has a key role in the identification of potential cancer biomarkers and this approach has been extensively used to discover CRC biomarkers [5]. The separation of protein mixtures by 1DE followed by separation of tryptic peptides by LC coupled to ESI-MS/MS with high mass resolution and accuracy served to identify proteins with high confidence and for label-free semi-quantitation by spectral counting [13].

To have predictive value, an ideal biomarker should be easy to measure and should detect the disease at a very early stage. Prospective studies are extremely important, since biomarkers can be discovered on samples collected years before the disease onset. Proteomics has seldom been employed to search for candidate biomarkers in plasma samples collected before CRC was diagnosed, and this sort of investigation has been reported only in women [14]. To the best of our knowledge, this is the first mass spectrometry-based proteomic study on a prospective investigation representative of the general population with the aim of discovering CRC biomarkers in blood. We focused on a CRC case–control study nested within the Florence cohort of the EPIC investigation. We have previously shown that human plasma samples currently in long-term storage in biobanks are amenable to omics analysis [15].

The study was preceded by an unbiased comprehensive analysis of the plasma proteome in a limited group of CRC patients enrolled at diagnosis and their matched controls. We then compared the results with those from an analogous global proteome analysis on a subpopulation of individuals from the EPIC cohort. In this early phase of the study we were interest in the identification of common changes in circulating protein profiles. To this end the differential proteome analyses were done on pooled samples, as this may minimize individual and technical variability while still maintaining the possibility of identifying changes induced by the disease, with the assumption that changes observed in pools correspond to the average of the individual changes [16,17].

Plasma proteins identified after the depletion of some high-abundance ones were still in the high to medium abundance range [18]. The initial exploratory phase was meant to identify proteins whose circulating levels changed in the presence of overt disease. The proteins identified were involved in inflammation (alpha-1-acid-glycoprotein, alpha-1-antichymotrypsin, C-reactive protein, C4b-binding protein, gelsolin, inter-alpha-trypsin inhibitor heavy chain H3) and immune response (C4b-binding protein, complement C5, galectin3-binding protein, vitamin K-dependent protein S), as suggested by systems biology tools and by a literature search [19-26]. This supports the notion that acute-phase proteins initiate or sustain inflammation, a process occurring in response to the presence of the tumour [19,23]. Altered plasma levels of some of these proteins have been reported for different tumour types, including colon and gastric [20,21].

Proteins involved in the immune response also showed altered levels, in agreement with evidence that an immune response is involved in CRC in addition to inflammation [27].

Plasma carbonic anhydrase 1 and peroxiredoxin-2 were lower in cases than controls, but because of their high abundance in red blood cells these proteins were not taken into account, since their presence in plasma might be due to haemolysis during blood collection [28].

Even though some plasma proteins identified in the exploratory study are different from those reported in earlier studies, the biological processes in which they are involved are essentially the same [29,30].

Comparison of the global plasma proteome of the exploratory and the EPIC studies indicated that most of the proteins identified were present in both studies, though in the EPIC there were fewer changes in the circulating protein levels. This comes as no surprise if we consider that the EPIC samples were collected several years before CRC diagnosis. However, PLS-DA analysis clearly distinguished EPIC CRC cases from controls and several proteins contributed to this result (proteins with VIP >1). Moreover, MetaCore enrichment analysis on proteins with changed levels indicated that the complement system cascade was the most significant process involved in both studies.

We validated proteins playing a major role in the separation of cases and controls in the EPIC cohort by targeted proteomics, a powerful technique allowing the quantitation of candidate biomarkers in complex mixtures across multiple samples with high selectivity and sensitivity [31]. Using a multiplexed LC-SRM-MS assay we assessed the relative amounts of all the CRC candidate biomarkers, including alpha-2-HS-glycoprotein (FETUA), an acute-phase response protein [19], apolipoprotein C-II (APOC2) involved in the catabolism of low- and high-density lipoproteins and inflammation [32], N-acetylmuramoyl-L-alanine amidase (PGRP2) belonging to the family of peptidoglycan recognition proteins of the innate immune system [33], complement C4-B (CO4-B), complement component C9 (CO9), CLU, mannan-binding lectin serine protease 2 (MASP2), and mannose-binding protein C (MBL2) involved in the complement cascade [23].

Targeted proteomics did not confirm the differences observed after global proteome analysis. The discrepancy is possibly due to the different sensitivity of the two analytical technologies, SRM-MS being more sensitive than MS/MS. Considering the whole EPIC population of our study, targeted proteomics indicated that CLU was the only protein slightly higher in CRC than in controls, but the difference was of borderline significance. This is in agreement with what was observed after the plasma global proteome analysis in the Phase 2 EPIC cohort, CLU showing FC = 1.32. Unlike in previous reports, we did not see any increase in circulating CLU in the Phase 1 exploratory study, possibly because of the limited number of individuals enrolled [34,35]. This does not depend on the analytical method, since absolute quantitation of plasma CLU showed concentrations in good agreement with reported data [36].

Interestingly, this study found that CLU was significantly higher in EPIC CRC males than in their matched controls. No such difference was seen in women. This was corroborated by further statistical analyses showing that the CLU ROC curve significantly distinguished male CRC cases from their matched controls. Furthermore, after multivariate adjustments, CLU was significantly associated with CRC only in men, with OR 1.8. This sex-related difference might not be a chance result, as other biomarker levels differ in men and women. We have previously shown in a large EPIC cohort that high circulating C-reactive protein, a marker of systemic inflammation, was related to colon cancer risk in men, but not in women [37]. More recently, the association of C-peptide, insulin, and insulin-like growth factor axis with colorectal carcinogenesis at an early stage was reported in men only [38].

The molecular basis for the sex difference is not known, but androgens might possibly be involved; an early study reported a higher incidence of chemically induced CRC in male than female rats, androgens being involved in this sex difference [39]. In addition, CLU expression in an androgen-dependent prostate cancer cell line was shown to increase in a time- and dose-dependent manner after androgen treatment both at mRNA and protein levels [40]. This effect was under the control of the androgen receptor (AR) and suggested that androgen regulation of CLU may be cytoprotective in the normal prostate [40]. AR signalling can be ligand-dependent or independent, the first pathway prevailing in men exposed to testicular androgens, and the second applies to both sexes. Though not proven by this study, different AR signalling in response to various stimuli might explain why CLU levels are higher in men than in women.

CLU is a chaperone ubiquitously expressed and involved in several physiological processes, but also in tumour growth and carcinogenesis [41,42]. CLU is an acute phase protein and a potent inhibitor of the terminal complement pathway, leading to reduced cytolysis and protection of the host cells from complement attack [43-45]. Nuclear and secreted isoforms of this protein are known whose function depends on the isoform involved, the nuclear isoform being pro-apoptotic and the secreted one cytoprotective [41,46-48]. In normal cells and early carcinogenesis CLU may inhibit tumour progression, whereas it may favour survival advantage in advanced tumours [42]. Increased CLU expression in tumour biopsies correlated with inhibition of apoptosis and tumour cell survival [41,49].

CLU has already been proposed as a diagnostic biomarker of CRC, based on analyses on samples collected at diagnosis [34,35]. This investigation shows for the first time that circulating CLU can be altered before the onset of the disease and suggests that plasma CLU measurements could be useful for identifying individuals at risk of developing CRC, at least among men.

It is not clear why plasma CLU increases in preclinical samples, but we can formulate some hypotheses. Since plasma samples were collected prospectively, the CLU changes might be related to the host response, rather than it being a cancer-derived biomarker. The intestine wall is protected by a mucosal barrier whose homeostasis is maintained by a multidimensional network, including commensal microbiota, host innate immunity and genetics [50]. Loss of balance of this physiological interaction might lead to inflammation and cancer and there is evidence that the intestinal microbiota plays a role in colorectal carcinogenesis [50,51]. Thus we can speculate that the increased circulating CLU in our population is likely to play a role in CRC development, since it might inhibit the host response to dangerous bacteria, thus allowing damage to the mucous intestinal barrier and favouring inflammation and cancer.

## Conclusions

The mass spectrometry-based analysis of the global plasma proteome identified, with high confidence, proteins involved in inflammation and/or immune processes, in samples collected at CRC diagnosis and years before it. In this preliminary study, the key finding is the identification of CLU as an early biomarker of CRC, at least in men. The main strengths of the study are the prospective design and the specificity of the analytical methods used to identify and validate candidate biomarkers. Though the small sample size is a limitation, the increased circulating CLU in preclinical samples warrants further investigation in a larger cohort of subjects to confirm the results of this pilot study and to assess the specificity of CLU as a biomarker for CRC, since it might help in identifying preventive intervention strategies.

## Protein name abbreviations

APOC2, apoliprotein C-II; CLU, clusterin; CO4-B, complement C4-B; CO9, complement component C9; FETUA, alpha-2-HS-glycoprotein (Fetuin A); MASP2, mannan-binding lectin serine-protease; MBL2, mannose-binding protein C; PGRP2, N-acetylmuramoyl-L-alanine amidase.

## Additional files

Additional file 1:
**Supplementary Methods.**

Additional file 2: Table S1. Phase 1 Exploratory Study, List of proteins identified by LC-ESI-MS/MS after 1DE separation and their relative quantitation by spectral counts. **Table S2.** Phase 2 EPIC Study, Proteins identified by LC-ESI-MS/MS after 1DE separation and their relative quantitation by spectral counts. **Table S3.** Relative amounts of proteins analysed by targeted proteomics (LC-SRM-MS) in the EPIC population not including Phase 2 samples (the number of subjects is given in parenthesis). **Table S4.** AUC of various combinations of protein biomarkers in males.
Additional file 3: Figure S1. PLS-DA analysis score scatter plot on proteins identified in the Phase 1 exploratory study after global proteome analysis. **Figure S2.** PLS-DA analysis score scatter plot on proteins identified in the Phase 2 EPIC study after global proteome analysis. **Figure S3.** Complement cascade: MetaCore “Network Enrichment analysis” on proteins with altered plasma levels (FC ≥ 1.5 or ≤ −1.5). **Figure S4.** SRM transition traces showing the separation of the selected peptides for the eight candidate biomarkers and the internal standard. **Figure S5.** Response linearity for the selected proteins after LC-SRM-MS analysis of increasing volumes of plasma.

## Abbreviations

1DE: One-dimensional gel electrophoresis
AR: Androgen receptor
CRC: Colorectal cancer
EPIC: European Prospective Investigation into Cancer and Nutrition
FC: Fold change
LC-ESI-MS/MS: Liquid chromatography-electrospray ionization-tandem mass spectrometry
LC-SRM-MS: Liquid chromatography-selected reaction monitoring-mass spectrometry
PLS-DA: Partial Least Squares-Discriminating Analysis
ROC: Receiver Operating Characteristic
VIP: Variable Importance in the Projection
