# Supplementary material for: Plasma clusterin as a candidate pre-diagnosis marker of colorectal cancer risk in the Florence cohort of the European Prospective Investigation into Cancer and Nutrition: a pilot study
Source: BMC Cancer. 2015 Feb 14;15:56. doi: 10.1186/s12885-015-1058-7 (PMC4334607; doi:10.1186/s12885-015-1058-7)
Supplement: Additional file 1: — Supplementary Methods. [file 12885_2015_1058_MOESM1_ESM.pdf]

SUPPLEMENTARY METHODS

Phase 1: Global proteome analysis

Sample preparation of the exploratory study. Fresh blood samples were collected using citrate as anticoagulant and immediately centrifuged at room temperature for 15 min at 1500*g* to separate plasma. Plasma proteome analysis was done on two pools obtained by mixing equal volumes of plasma from ten healthy subjects and ten CRC cases.

Six of the most abundant plasma proteins, namely serum albumin, IgG, IgA, transferrin, haptoglobin, antitrypsin, were depleted using the multiple affinity removal system (MARS, Agilent Technologies) according to the manufacturer's protocol. The affinity column (4.6 mm I.D., x 50 mm long) contains immobilized polyclonal antibodies raised against the six proteins. The reagent kit (Agilent Technologies) included proprietary Buffer A for sample loading, column washing and regenerating, and Buffer B for the elution of bound proteins. Briefly, human plasma was diluted 1:5 (v:v) with Buffer A with a protease inhibitor cocktail added (Complete Mini, EDTA free, Roche Diagnostic Corporation). After filtration through a 0.22 µm spin filter at 16,000xg for 2 min at room temperature, the diluted plasma sample (70 µL, equivalent to 14 µL crude plasma) was injected into the MARS column connected to a Perkin Elmer series 200 HPLC, with a mobile phase flow rate of 250 µL/min. Flow-through low-abundance proteins were monitored at 280 nm. The linear solvent gradient was as follows: from 100% Buffer A to 100% Buffer B in 9 min, then 100% Buffer B kept for 3.5 min before column re-equilibration to 100% Buffer A for 7.5 min. Flow-through fraction was collected between 1.9-6.4 min (1.5 mL) and added drop wise to absolute cold ethanol, under mixing, to a final ethanol concentration of 90%, for protein precipitation and desalting. After centrifugation at 9400xg for 15 min at 4°C, the protein pellets were dissolved in 300 µL rehydration buffer (5M urea, 2M thiourea, 2% CHAPS, 2% Zwittergent), and the protein concentration of each samples was measured using the BioRad assay (Bio-Rad, Milan, Italy).

One-dimensional gel electrophoresis (1DE). Triplicates of pooled plasma samples (20 µg/replicate) were mixed with an equal volume of loading buffer (100 mM Tris-HCl pH 6.8, 4% SDS, 20% glycerol, 0.2% bromophenol blue, 100 mM dithithreitol, DTT) heated at 37°C for 15 min and resolved on precast Nupage 4-12% Bis-Tris gel 1.0 mm x 10 wells, 8x8 cm (Invitrogen) at 200V, for about 50 min. Gels were rinsed three times with de-ionized H_2O, fixed for 1 h in an aqueous solution with 50% methanol and 7% acetic acid, and rinsed again with de-ionized H_2O. Finally, gels were stained overnight with colloidal Coomassie Blue (CCB, Pierce, Rockford, IL) then extensively washed with de-ionized H_2O. After staining, each gel lane was manually cut with a sterile surgical blade into 24 slices of equal height (about 3 mm). Each slice was placed in an Eppendorf tube, crushed into very small pieces, and trypsin-digested [1]. To maximise peptide recovery, after collecting the peptide-rich supernatant, the gel plugs were extracted twice (37°C, 15 min) with 60 µL and 40 µL acetonitrile. The two peptide extracts were pooled, dried and redissolved in the previously collected supernatant (20 µL). The final sample thus contained all peptides recovered from the digestion of a single gel slice [1].

Protein identification after 1DE. Two µL of each sample were directly analyzed by LC-ESI-MS/MS, with an LTQ Orbitrap XL (Thermo Scientific, Waltham, MA) interfaced with a 1200 series capillary pump (Agilent, Santa Clara, CA). Peptides were separated on a Thermo Scientific Biobasic 18 column (150 × 0.18 mm ID, particle size 5 µm). Operating conditions were as reported by Schiarea et al. 2010 [1] with minor modifications. Briefly, LC conditions were: column flow 2 µL/min; eluant A, H_2O and 0.1% formic acid; eluant B, acetonitrile; gradient program, from 2% to 60% B in 35 min, then to 98% B in 11 min, kept there for 4 min, then returned to 2% B in 2 min and kept at 2% B for re-equilibration for 35 min. MS conditions were as reported in Schiarea et al, 2010 [1].

All individual MS/MS spectra in an LC run were exported into dta files by Bioworks browser 3.3.1 (Thermo Scientific, Waltham, MA). MS/MS spectra from the same precursor ion were grouped using a tolerance of 2 ppm for MS/MS spectra with at least 100-counts intensity and at least 10 ions, with automatic assignment of charge state.

Dta files from the 24 gel slices were merged and submitted as an mgf file to the search engines Mascot (in-house version 2.2, Matrix Science, Boston, MA) and Scaffold (version 3_00_02, Proteome Software Inc., Portland, OR, US), the latter used to further validate MS/MS-based peptide and protein identifications generated by Mascot. Scaffold combines data from Mascot and X!Tandem, another search engine that works on a different algorithm. Mascot and Scaffold searches were done against the Swiss-Prot UniProt database, version 56.5. Search parameters were: "*Homo sapiens*" taxonomy; no restriction on molecular weight (MW); enzyme, trypsin (one missed cleavage allowed); fixed modification, carbamidomethylation of cysteine; variable modification, oxidation of methionine; experimental mass values, monoisotopic; peptide mass tolerance, 2 ppm; MS/MS mass tolerance, 1 Da; peptide charge, 2+, 3+, 4+; decoy search, active.

Peptide identifications were accepted if they could be established at greater than 95.0% probability as specified by the Peptide Prophet algorithm [2]. Protein identifications were accepted if established at greater than 99.9% probability with at least two non-redundant identified peptides. Protein probabilities were assigned by the Protein Prophet algorithm [3]. These filtering criteria gave a false positive identification rate (FDR) of 0%.

Scaffold allows the label-free relative quantitation of identified proteins based on normalized spectral counts across each experiment. Protein identification was considered valid with a minimum of three spectral counts/replicate in at least one group. To ensure the correct identification of proteins identified with only two non-redundant peptides and a low number of spectral counts, the peptide MS/MS spectra were manually inspected. For each identified protein, the number of spectral counts was exported to Excel and normalized counts were averaged for replicate samples. Subsequently the estimation of

differential protein abundance was expressed as the –fold change (FC, ratio of the averaged spectral counts in the CRC samples to the averaged spectral counts in the control samples).

Phase 2: Global proteome analysis

Sample preparation of the EPIC study. The blood samples used in this study were collected and stored according to the standardized international protocol used in EPIC study [4]. Blood samples were aliquoted in the same day of the collection into 28 plastic straws containing 0.5 ml each (12 plasma, eight serum, four erythrocytes, four buffy coat for DNA). After a rapid cooling in freezer at -80°C, the straws of each participant were stored in liquid nitrogen at -196°C together successively inside a tube, goblet, canister and container. Each straw was labelled with the participant's ID and colour-coded to indicate its contents. A detailed software program has been developed to recover the exact position of each sample.

Ten controls and ten individuals who developed CRC during the follow-up were selected and two pools were created by mixing equal volumes of plasma. High-abundant proteins were depleted using MARS spin cartridges (Agilent Technologies) according to the manufacturer's protocol. This depletion system has two advantages compared with the MARS Column used for Phase 1 plasma samples: (i) in addition to the six proteins removed with the MARS column, it removes fibrinogen too, and (ii) it is less time-consuming, because of the use of a micro-centrifuge instead of an HPLC run.

Depleted plasma samples were desalted and proteins concentrated as for Phase 1 samples. The protein pellets were dissolved in 90 µL rehydration buffer (5M urea, 2M thiourea, 2% CHAPS, 2% Zwittergent), and the protein concentration of each sample was determined using the BioRad assay (Bio-Rad, Milan, Italy). Proteins (20 µg, three replicate lanes/pool) were separated by 1DE and identified by LC-ESI-MS/MS as described above.

Phase 3: Candidate biomarker validation in the EPIC population

In this phase eight candidate biomarkers identified in Phase 2 were analysed in the 96 samples of the EPIC-Florence cohort by targeted mass spectrometry. Except for the 20 samples used in Phase 2, the control/case status of the remaining 76 samples was not known to the laboratory, as the other demographic characteristics of the study cohort. The technique used was Selected Reaction Monitoring coupled to liquid chromatography (LC-SRM-MS), a highly specific and sensitive non-scanning mass spectrometry technique, able to quantify compounds within complex mixtures, such as plasma. This procedure involves the measurement of a given peptide formed in stoichiometric quantities from the protein of interest after proteolytic digestion, thus allowing the quantitation of the original protein [5].

The LC-SRM-MS experiment involves different steps: (i) selection of candidate biomarker proteins; (ii) definition of the most representative peptides (proteotypic peptides) of a given protein; (iii) selection of transitions; (iv) optimization of the experimental parameter, such as collision energy (CE); (v) choice of internal standard; (vi) LC-SRM-MS. The choice of candidate biomarkers was based on the results from global proteome analysis, and from multivariate and system biology analyses. Tandem mass spectrometry data from global proteomic analysis were investigated for the selection of proteotypic peptides, and relative transitions. After preliminary experiment (not shown), we selected the proteotypic peptide giving the best response for each protein. The following restriction criteria were adopted: number of amino acid residues ≥ 8, double charge, no missed cleavage, lack of methionine, tryptophan and cystein residues. The best transitions were confirmed from specific databases, such as “PeptideAtlas” (www.peptideatlas.org) and “The Global Proteome Machine” (www.thegpm.org). Energy collision for SRM analysis was set experimentally. We selected bovine fetuin as internal standard (IS), a protein spiked with a known amount in the sample and used for protein quantitation. We considered a peptide not present in the human fetuin [6].

In-solution trypsin digestion of plasma samples and standard proteins. A new method for in-solution trypsin digestion of plasma proteins was set up, that does not require the depletion of high-abundant proteins. After centrifugation of all 96 plasma samples at 16000xg for 2 min, 5 µL of plasma were taken from each sample and mixed with 5 µL of rehydration buffer (5 M urea, 2 M thiourea, 2% CHAPS, 2% Zwittergent), 38.1 µL H_2O, and 24.8 µL 1 M ammonium bicarbonate (AmBic). Plasma proteins were reduced with 2 µL 100 mM DTT in 0.1 M AmBic at 56°C for 1 h, and alkylated with 2 µL 270 mM iodoacetamide (IAA) in 0.1 M AmBic for 30 min, at room temperature, in the dark. To maintain trypsin activity, the urea concentration was brought to 0.1 M by adding 165 µL 100 mM Ambic. Seven µg sequencing-grade trypsin (Roche, Mannheim, Germany) were added to the samples (w/w enzyme/protein = 1/50) and digestion was at 37°C overnight. The digestion was stopped by acidifying the samples with 1% acetic acid (5 µL).

Four pmol of IS, digested separately, were added to each digested plasma sample and the mixtures were acidified with 1% formic acid (200 µL) to a final pH <4. To concentrate digested plasma samples and eliminate salts and denaturing agents that could interfere with LC-SRM-MS analysis we did solid phase extraction (SPE) using Isolute C18 1 mg/mL columns (Biotage, Uppsala, Sweden). Before loading the samples, columns were washed with 2 x 1 mL of acetonitrile/H_2O (1/1) then equilibrated with 2 x 1 mL 0.1% formic acid in H_2O. Acidified samples (final volume 465 µL) were loaded onto the columns and flowed through. Columns were then washed with 2 x 1 mL 0.1% formic acid in H_2O to remove salts and impurities, bound fractions were eluted with 200 µL of a 1/1 solution of acetonitrile/0.1% formic acid in H_2O. The eluates were concentrated to a final volume of 20 µL using the Concentrator 5301 (Eppendorf).

After SPE desalting and concentration, the 96 EPIC samples were analysed for the relative quantitation of the selected proteins.

A 1200 series capillary pump (Agilent, Santa Clara, CA) coupled to an Agilent 6410 triple quadrupole mass spectrometer with an electrospray ionisation source was used. Peptides were separated on a Chromolith RP C18 50x2 mm column (Merck, Germany). LC conditions were: mobile phase flow 0.2 mL/min; solvent A, 0.1% formic acid in H_2O; solvent B, acetonitrile; gradient program, from 2% to 45% B in 12 min, then to 98% B in 1 min, kept there for 2 min, then returned to initial conditions in 10 min. Peptides were ionised at 4.5 kV, the spray gas temperature was 300°C, the gas flow was 8 L/min, gas pressure was 45 psi. Data were acquired with 50 ms dwell time. The chromatographic run was divided into four time segments with the mass spectrometer set to scan different transitions during each time fragment according to the peptide retention times.

One peptide was selected for each protein, two transitions per peptide were monitored, one used for quantitation and the other to maximise specificity. The relative quantitation was the ratio of the peak area of the analyte to that of the IS. Peptides with peak area below 1000 (3/1 = signal/noise) were considered not valid. Triplicate LC-SRM-MS analyses were run on all EPIC-Florence plasma samples.

Response linearity for the selected peptides was assessed by analysing increasing amounts of the pooled plasma samples previously analysed for global proteome characterisation. Briefly, 0.5, 1, 2, 4 μL plasma underwent in-solution trypsin digestion as above and LC-SRM-MS analysis.

Calibration curves for the absolute quantitation of Clusterin (CLU) were plotted using increasing amounts of standard protein (BioVendor, Germany). Briefly, 0.2, 0.4, 0.8, 1.6, 3.2 pmol CLU were trypsin-digested as above and analysed by LC-SRM-MS.

Functional and Pathway analysis

MetaCore version 6.12 (GeneGo, St Joseph, MI, USA) was used to map the differentially expressed proteins into biological networks and for functional interpretation of the protein data. MetaCore is an integrated software suite based on a manually curated database of

human/mouse/rat protein-protein interactions, protein-DNA interactions, transcriptional factors, metabolic and signalling pathways. A list of proteins with circulating levels 1.5 times higher or lower than controls was prepared for Phase 1 and 2 studies and uploaded as their Swiss-Prot identifiers to MetaCore for analysis against the default background (i.e. the entire MetaCore database), setting the significance threshold for the FDR filter at p <0.05. The Enrichment Analysis Workflow was used to compare experimental data by analysing their mapping onto MetaCore's various ontologies, including GenoGo Pathway Maps, GenoGo Process Networks, Diseases, and GO Processes. The statistical scores throughout MetaCore are calculated using a hypergeometric distribution, where the *p* value represents the probability that a process appears in the data set relative to that expected by chance. The sorting method "Statistically significant" was chosen, which calculates the maximum -log(pValue) for every ontology term and sorts the whole ontology in decreasing order. The top few terms are displayed as histograms where each histogram section corresponds to a specific term of the ontology, graphically representing the -log(pValue) of the mapping of the experiment onto that term.

References

1. Schiarea S, Solinas G, Allavena P, Scigliuolo GM, Bagnati R, Fanelli R, Chiabrando C: **Secretome analysis of multiple pancreatic cancer cell lines reveals perturbations of key functional networks**. *J Proteome Res* 2010, **9**:4376-4392.
2. Keller A, Nesvizhskii AI, Kolker E, Aebersold R: **Empirical statistical model to estimate the accuracy of peptide identifications made by MS/MS and database search**. *Anal Chem* 2002, **74**:5383-5392.
3. Nesvizhskii AI, Keller A, Kolker E, Aebersold R: **A statistical model for identifying proteins by tandem mass spectrometry**. *Anal Chem* 2003, **75**:4646-4658.

4. Riboli E, Hunt KJ, Slimani N, Ferrari P, Norat T, Fahey M, Charrondiere UR, Hemon B, Casagrande C, Vignat J, Overvad K, Tjonneland A, Clavel-Chapelon F, Thiebaut A, Wahrendorf J, Boeing H, Trichopoulos D, Trichopoulou A, Vineis P, Palli D, Bueno-De-Mesquita HB, Peeters PH, Lund E, Engeset D, Gonzalez CA, Barricarte A, Berglund G, Hallmans G, Day NE, Key TJ *et al.*: **European Prospective Investigation into Cancer and Nutrition (EPIC): study populations and data collection**. *Public Health Nutr* 2002, **5**:1113-1124.
5. Lange V, Picotti P, Domon B, Aebersold R: **Selected reaction monitoring for quantitative proteomics: a tutorial**. *Mol Syst Biol* 2008, **4**:222.
6. Yang Z, Hayes M, Fang X, Daley MP, Ettenberg S, Tse FL: **LC-MS/MS Approach for Quantification of Therapeutic Proteins in Plasma Using a Protein Internal Standard and 2D-Solid-Phase Extraction Cleanup**. *Anal Chem* 2007, **79**:9294-9301.
