# Supplementary material for: Plasma clusterin as a candidate pre-diagnosis marker of colorectal cancer risk in the Florence cohort of the European Prospective Investigation into Cancer and Nutrition: a pilot study
Source: BMC Cancer. 2015 Feb 14;15:56. doi: 10.1186/s12885-015-1058-7 (PMC4334607; doi:10.1186/s12885-015-1058-7)
Supplement: Additional file 2: Table S1. — Phase 1 Exploratory Study, List of proteins identified by LC-ESI-MS/MS after 1DE separation and their relative quantitation by spectral counts. Table S2. Phase 2 EPIC Study, Proteins identified by LC-ESI-MS/MS after 1DE separation and their relative quantitation by spectral counts. Table S3. Relative amounts of proteins analysed by targeted proteomics (LC-SRM-MS) in the EPIC population not including Phase 2 samples (the number of subjects is given in parenthesis). Table S4. AUC of various combinations of protein biomarkers in males. [file 12885_2015_1058_MOESM2_ESM.pdf]

Table S1.

Phase 1 Exploratory Study, List of proteins identified by LC-ESI-MS/MS after 1DE separation and their relative quantitation by spectral count

Protein Name	UniProt Entry Name	SwissProt-UniProt No.	CRC case Pool			Control Pool			FC [b]	VIP value[d]
			Spectral Counts Mean ± SD [a]			Spectral Counts Mean ± SD [a]				
Actin, cytoplasmic 1	ACTB_HUMAN	P60709	5	±	2	6	±	7	-1.27	0.1668
Afamin	AFAM_HUMAN	P43652	23	±	1	30	±	7	-1.30	0.7291
Alpha-1-acid glycoprotein	A1AG1_HUMAN	P02763	6	±	1	3	±	0.4	**1.85**	0.5556
Alpha-1-acid glycoprotein 2	A1AG2_HUMAN	P19652	3	±	2	3	±	1	-1.00	0.0011
Alpha-1-antichymotrypsin	AACT_HUMAN	P01011	100	±	8	43	±	8	**2.32**	**2.5592**
Alpha-1B-glycoprotein	A1BG_HUMAN	P04217	20	±	5	20	±	7	-1.04	0.0912
Alpha-2-antiplasmin	A2AP_HUMAN	P08697	14	±	3	12	±	2	1.19	0.3603
Alpha-2-HS-glycoprotein	FETUA_HUMAN	P02765	19	±	6	27	±	7	-1.43	0.7309
Alpha-2-macroglobulin	A2MG_HUMAN	P01023	527	±	46	689	±	73	-1.31	**4.0391**
Angiotensinogen	ANGT_HUMAN	P01019	13	±	1	14	±	6	-1.02	0.0353
Antithrombin-III	ANT3_HUMAN	P01008	57	±	10	62	±	14	-1.07	0.3285
Apolipoprotein A-I	APOA1_HUMAN	P02647	195	±	32	226	±	26	-1.16	**1.4098**
Apolipoprotein A-II	APOA2_HUMAN	P02652	15	±	1	18	±	6	-1.19	0.3571
Apolipoprotein A-IV	APOA4_HUMAN	P06727	37	±	7	47	±	7	-1.27	0.8728
Apolipoprotein B-100	APOB_HUMAN	P04114	467	±	84	475	±	78	-1.02	0.2437
Apolipoprotein D	APOD_HUMAN	P05090	26	±	9	25	±	6	1.03	0.0643
Apolipoprotein E	APOE_HUMAN	P02649	39	±	5	34	±	7	1.13	0.4459
Apolipoprotein L1	APOL1_HUMAN	O14791	10	±	1	10	±	2	-1.05	0.1040
Apolipoprotein M	APOM_HUMAN	O95445	8	±	2	6	±	2	1.36	0.3465
Beta-2-glycoprotein 1	APOH_HUMAN	P02749	30	±	7	26	±	4	1.15	0.4219
C-reactive protein	CRP_HUMAN	P02741	12	±	1	1	±	1	**16.97**	**1.1243**
C4b-binding protein alpha chain	C4BPA_HUMAN	P04003	53	±	6	35	±	3	**1.51**	**1.3888**
Carbonic anhydrase 1	CAH1_HUMAN	P00915	3	±	1	8	±	1	***-2.48***	0.7014
Carboxypeptidase N catalytic chain	CBPN_HUMAN	P15169	6	±	1	6	±	4	1.10	0.0930
Carboxypeptidase N subunit 2	CPN2_HUMAN	P22792	4	±	2	5	±	1	-1.05	0.0412
CD5 antigen-like	CD5L_HUMAN	O43866	3	±	1	5	±	2	***-1.72***	0.4023
Ceruloplasmin	CERU_HUMAN	P00450	123	±	21	106	±	20	1.16	0.9296
Clusterin	CLUS_HUMAN	P10909	31	±	3	33	±	4	-1.07	0.2766

continued Table S1.

Protein Name	UniProt Entry Name	SwissProt-UniProt No.	CRC case Pool			Control Pool			FC [b]	VIP value[d]
			Spectral Counts Mean ± SD [a]			Spectral Counts Mean ± SD [a]				
Coagulation factor X	FA10_HUMAN	P00742	3	±	2	4	±	4	-1.12	0.0643
Coagulation factor XII	FA12_HUMAN	P00748	4	±	1	3	±	1	1.11	0.1111
Complement C1q subcomponent subunit B	C1QB_HUMAN	P02746	10	±	4	9	±	2	1.07	0.1069
Complement C1q subcomponent subunit C	C1QC_HUMAN	P02747	14	±	4	9	±	1	**1.57**	0.6476
Complement C1r subcomponent	C1R_HUMAN	P00736	10	±	4	8	±	1	1.24	0.3002
Complement C1s subcomponent	C1S_HUMAN	P09871	15	±	1	19	±	3	-1.27	0.5884
Complement C2	CO2_HUMAN	P06681	14	±	5	12	±	4	1.17	0.2675
Complement C3	CO3_HUMAN	P01024	837	±	34	773	±	106	1.08	**1.8297**
Complement C4-B	CO4B_HUMAN	P0C0L5	218	±	8	185	±	35	1.18	**1.5759**
Complement C5	CO5_HUMAN	P01031	64	±	2	41	±	6	**1.55**	**1.6023**
Complement component C6	CO6_HUMAN	P13671	18	±	3	13	±	1	1.38	0.7032
Complement component C7	CO7_HUMAN	P10643	11	±	2	12	±	5	-1.18	0.2555
Complement component C8 alpha chain	CO8A_HUMAN	P07357	5	±	1	4	±	1	1.21	0.2479
Complement component C8 beta chain	CO8B_HUMAN	P07358	6	±	1	4	±	3	1.29	0.2081
Complement component C8 gamma chain	CO8G_HUMAN	P07360	14	±	2	10	±	3	1.38	0.5522
Complement component C9	CO9_HUMAN	P02748	18	±	5	11	±	5	**1.60**	0.7036
Complement factor B	CFAB_HUMAN	P00751	80	±	12	55	±	6	1.44	**1.5539**
Complement factor H	CFAH_HUMAN	P08603	79	±	18	81	±	3	-1.02	0.1057
Complement factor H-related protein 1	FHR1_HUMAN	Q03591	13	±	2	11	±	4	1.11	0.1976
Complement factor I	CFAI_HUMAN	P05156	15	±	1	10	±	1	1.40	0.6639
Corticosteroid-binding globulin	CBG_HUMAN	P08185	6	±	1	6	±	1	1.09	0.1445
Fibrinogen alpha chain	FIBA_HUMAN	P02671	556	±	23	403	±	66	1.38	**3.9906**
Fibrinogen beta chain	FIBB_HUMAN	P02675	277	±	20	161	±	30	**1.72**	**3.5866**
Fibrinogen gamma chain	FIBG_HUMAN	P02679	213	±	29	152	±	25	1.40	**2.4133**
Fibronectin	FINC_HUMAN	P02751	118	±	8	110	±	13	1.07	0.5892
Fibulin-1	FBLN1_HUMAN	P23142	2	±	1	4	±	0.4	***-2.33***	0.4732
Ficolin-3	FCN3_HUMAN	O75636	6	±	1	4	±	1	**1.60**	0.4612
Galectin-3-binding protein	LG3BP_HUMAN	Q08380	3	±	2	9	±	1	***-3.18***	0.8048
Gelsolin	GELS_HUMAN	P06396	23	±	6	39	±	6	***-1.67***	**1.2344**

continued Table S1. page 3/4

Protein Name	UniProt Entry Name	SwissProt-UniProt No.	CRC case Pool			Control Pool			FC [b]	VIP value[d]
			Spectral Counts Mean ± SD [a]			Spectral Counts Mean ± SD [a]				
Glutathione peroxidase 3	GPX3_HUMAN	P22352	4	±	0.2	3	±	0.3	1.37	0.3465
Hemopexin	HEMO_HUMAN	P02790	60	±	15	65	±	15	-1.09	0.3678
Heparin cofactor 2	HEP2_HUMAN	P05546	18	±	3	15	±	4	1.17	0.3433
Histidine-rich glycoprotein	HRG_HUMAN	P04196	18	±	1	19	±	1	-1.06	0.2602
Ig kappa chain C region	IGKC_HUMAN	P01834	10	±	1	8	±	3	1.21	0.2959
Ig lambda-2 chain C regions	LAC2_HUMAN	P0CG05	13	±	2	12	±	3	1.05	0.0953
Ig mu chain C region	IGHM_HUMAN	P01871	20	±	0	22	±	1	-1.10	0.4196
Insulin-like growth factor-binding protein complex acid labile subunit	ALS_HUMAN	P35858	6	±	3	7	±	2	-1.07	0.0715
Inter-alpha-trypsin inhibitor heavy chain H1	ITIH1_HUMAN	P19827	67	±	6	55	±	7	1.21	**0.9998**
Inter-alpha-trypsin inhibitor heavy chain H2	ITIH2_HUMAN	P19823	145	±	9	124	±	28	1.17	**1.1418**
Inter-alpha-trypsin inhibitor heavy chain H3	ITIH3_HUMAN	Q06033	11	±	0	4	±	1	**2.61**	0.8991
Inter-alpha-trypsin inhibitor heavy chain H4	ITIH4_HUMAN	Q14624	62	±	5	63	±	6	-1.02	0.1463
Kallistatin	KAIN_HUMAN	P29622	1	±	1	7	±	3	***-5.44***	0.7907
Kininogen-1	KNG1_HUMAN	P01042	31	±	1	34	±	9	-1.09	0.3042
Leucine-rich alpha-2-glycoprotein	A2GL_HUMAN	P02750	15	±	6	7	±	3	**1.99**	0.7601
Mannose-binding protein C	MBL2_HUMAN	P11226	4	±	1	2	±	1	**1.81**	0.3600
N-acetylmuramoyl-L-alanine amidase	PGRP2_HUMAN	Q96PD5	4	±	0.5	5	±	3	-1.28	0.1984
Peroxiredoxin-2	PRDX2_HUMAN	P32119	3	±	1	5	±	1	***-1.83***	0.4945
Pigment epithelium-derived factor	PEDF_HUMAN	P36955	14	±	4	16	±	5	-1.18	0.3007
Plasma kallikrein	KLKB1_HUMAN	P03952	18	±	2	22	±	5	-1.20	0.4766
Plasma protease C1 inhibitor	IC1_HUMAN	P05155	36	±	8	36	±	4	1.02	0.0688
Plasminogen	PLMN_HUMAN	P00747	39	±	6	34	±	5	1.14	0.5118
Protein AMBP	AMBP_HUMAN	P02760	25	±	3	22	±	3	1.13	0.4061
Prothrombin	THRB_HUMAN	P00734	35	±	9	31	±	9	1.12	0.3236
Retinol-binding protein 4	RET4_HUMAN	P02753	26	±	6	27	±	5	-1.04	0.1281
Serum amyloid A protein	SAA_HUMAN	P0DJI8	8	±	0.3	nd[c]			nd[c]	0.9784
Serum amyloid A-4 protein	SAA4_HUMAN	P35542	18	±	1	14	±	2	1.30	0.6385
Serum amyloid P-component	SAMP_HUMAN	P02743	17	±	1	15	±	6	1.16	0.2992

continued Table S1. page4/4 **page 4/4**

Protein Name	UniProt Entry Name	SwissProt-UniProt No.	CRC case Pool			Control Pool			FC [b]	VIP value [d]
			Spectral Counts Mean ± SD [a]			Spectral Counts Mean ± SD [a]				
Serum paraoxonase/arylesterase 1	PON1_HUMAN	P27169	18	±	4	22	±	3	-1.22	0.5052
Sex hormone-binding globulin	SHBG_HUMAN	P04278	2	±	1	3	±	2	***-1.69***	0.2730
Tetranectin	TETN_HUMAN	P05452	5	±	3	6	±	2	-1.11	0.0960
Thyroxine-binding globulin	THBG_HUMAN	P05543	6	±	2	7	±	1	-1.00	0.0026
Transthyretin	TTHY_HUMAN	P02766	50	±	7	57	±	8	-1.13	0.6127
Vitamin D-binding protein	VTDB_HUMAN	P02774	51	±	4	57	±	9	-1.12	0.6018
Vitamin K-dependent protein S	PROS_HUMAN	P07225	6	±	1	4	±	1	**1.76**	0.5437
Vitronectin	VTNC_HUMAN	P04004	20	±	4	17	±	1	1.17	0.4184
Zinc-alpha-2-glycoprotein	ZA2G_HUMAN	P25311	9	±	2	10	±	1	-1.08	0.1432

a Mean ± SD of three technical replicates

[b] FC, Fold Change, Spectral counts of CRC cases/controls; **bold**, FC≥1.5; ***bold-italic***, FC≤-1.5

[c] nd, not detectable

[d] VIP, Variable Importance in the Projection (PLS-DA analysis); **bold,** VIP>1

Table S2.

Phase 2 EPIC Study, Proteins identified by LC-ESI-MS/MS after 1DE separation and their relative quantitation by spectral count

Protein Name	UniProt Entry Name	SwissProt-UniProt No.	EPIC-CRC case Pool			EPIC-Control Pool			FC[b]	VIP value[c]
			Spectral Counts Mean ± SD [a]			Spectral Counts Mean ± SD [a]				
Actin, cytoplasmic 1	ACTB_HUMAN	P60709	11	±	8	8	±	1	**1.50**	0.5594
Adiponectin	ADIPO_HUMAN	Q15848	4	±	3	6	±	3	***-1.57***	0.4922
Afamin	AFAM_HUMAN	P43652	56	±	9	46	±	10	1.22	**1.1485**
Alpha-1-acid glycoprotein 1	A1AG1_HUMAN	P02763	115	±	53	144	±	8	-1.25	**1.6894**
Alpha-1-acid glycoprotein 2	A1AG2_HUMAN	P19652	69	±	27	85	±	12	-1.23	**1.2329**
Alpha-1-antichymotrypsin	AACT_HUMAN	P01011	230	±	10	227	±	43	1.01	0.2015
Alpha-1B-glycoprotein	A1BG_HUMAN	P04217	59	±	27	53	±	6	1.11	0.4853
Alpha-2-antiplasmin	A2AP_HUMAN	P08697	38	±	18	36	±	11	1.07	0.2540
Alpha-2-HS-glycoprotein	FETUA_HUMAN	P02765	142	±	24	162	±	21	-1.14	**1.4575**
Alpha-2-macroglobulin	A2MG_HUMAN	P01023	1023	±	95	1041	±	38	-1.02	0.8031
Angiotensinogen	ANGT_HUMAN	P01019	41	±	8	42	±	2	-1.03	0.1725
Antithrombin-III	ANT3_HUMAN	P01008	115	±	42	156	±	36	-1.36	**2.2855**
Apolipoprotein A-I	APOA1_HUMAN	P02647	549	±	138	780	±	256	-1.42	**5.5626**
Apolipoprotein A-II	APOA2_HUMAN	P02652	32	±	16	16	±	2	**1.98**	**1.5656**
Apolipoprotein A-IV	APOA4_HUMAN	P06727	152	±	22	150	±	13	1.02	0.2208
Apolipoprotein B-100	APOB_HUMAN	P04114	770	±	41	826	±	92	-1.07	**2.4101**
Apolipoprotein C-II	APOC2_HUMAN	P02655	7	±	2	3	±	2	**2.44**	0.9406
Apolipoprotein D	APOD_HUMAN	P05090	52	±	8	50	±	2	1.03	0.2718
Apolipoprotein E	APOE_HUMAN	P02649	77	±	17	72	±	14	1.08	0.5339
Apolipoprotein L1	APOL1_HUMAN	O14791	24	±	5	23	±	9	1.05	0.1499
Apolipoprotein M	APOM_HUMAN	O95445	14	±	2	13	±	3	1.01	0.0182
Apolipoprotein(a)	APOA_HUMAN	P08519	8	±	3	7	±	0	1.18	0.2993
Attractin	ATRN_HUMAN	O75882	5	±	1	9	±	2	***-1.86***	0.8809

continued Table S2.

Protein Name	UniProt Entry Name	SwissProt-UniProt No.	EPIC-CRC case Pool			EPIC-Control Pool			FC[b]	VIP value[c]
			Spectral Counts Mean ± SD [a]			Spectral Counts Mean ± SD [a]				
Beta-2-glycoprotein 1	APOH_HUMAN	P02749	38	±	8	45	±	5	-1.19	0.9607
Beta-Ala-His dipeptidase	CNDP1_HUMAN	Q96KN2	6	±	2	3	±	2	**1.70**	0.6108
C4b-binding protein alpha chain	C4BPA_HUMAN	P04003	12	±	5	12	±	1	-1.04	0.0923
Carbonic anhydrase 1	CAH1_HUMAN	P00915	3	±	1	1	±	0	**2.89**	0.6302
Carboxypeptidase B2	CBPB2_HUMAN	Q96IY4	4	±	2	6	±	1	***-1.72***	0.6387
Carboxypeptidase N catalytic chain	CBPN_HUMAN	P15169	11	±	3	11	±	4	-1.03	0.0560
Carboxypeptidase N subunit 2	CPN2_HUMAN	P22792	11	±	5	9	±	3	1.19	0.3182
CD5 antigen-like	CD5L_HUMAN	O43866	5	±	2	6	±	1	-1.07	0.1233
Ceruloplasmin	CERU_HUMAN	P00450	267	±	85	222	±	25	1.20	**2.0454**
Clusterin	CLUS_HUMAN	P10909	64	±	13	48	±	4	1.32	**1.6267**
Coagulation factor X	FA10_HUMAN	P00742	8	±	2	8	±	3	1.03	0.0526
Coagulation factor XII	FA12_HUMAN	P00748	8	±	2	6	±	1	1.45	0.6276
Coagulation factor XIII A chain	F13A_HUMAN	P00488	7	±	0	6	±	1	1.23	0.4378
Coagulation factor XIII B chain	F13B_HUMAN	P05160	4	±	2	2	±	1	**1.85**	0.4534
Complement C1q subcomponent subunit A	C1QA_HUMAN	P02745	33	±	4	32	±	5	1.03	0.1455
Complement C1q subcomponent subunit B	C1QB_HUMAN	P02746	25	±	4	23	±	4	1.09	0.3633
Complement C1q subcomponent subunit C	C1QC_HUMAN	P02747	26	±	1	25	±	3	1.02	0.1185
Complement C1r subcomponent	C1R_HUMAN	P00736	18	±	5	19	±	9	-1.07	0.1707
Complement C1s subcomponent	C1S_HUMAN	P09871	46	±	22	30	±	5	**1.52**	**1.3905**
Complement C2	CO2_HUMAN	P06681	13	±	8	15	±	4	-1.16	0.3140
Complement C3	CO3_HUMAN	P01024	1451	±	179	1504	±	110	-1.04	**1.6302**
Complement C4-B	CO4B_HUMAN	P0C0L5	445	±	86	411	±	37	1.08	**1.5668**
Complement C5	CO5_HUMAN	P01031	88	±	6	92	±	20	-1.05	0.4390
Complement component C6	CO6_HUMAN	P13671	24	±	4	27	±	7	-1.13	0.4751
Complement component C7	CO7_HUMAN	P10643	10	±	5	16	±	1	***-1.64***	**1.0388**
Complement component C8 alpha chain	CO8A_HUMAN	P07357	17	±	2	14	±	3	1.16	0.5004
Complement component C8 beta chain	CO8B_HUMAN	P07358	22	±	3	17	±	12	1.31	0.6669

continued Table S2.										page 3/5
Protein Name	UniProt Entry Name	SwissProt-UniProt No.	EPIC-CRC case Pool			EPIC-Control Pool			FC[b]	VIP value[c]
			Spectral Counts Mean ± SD [a]			Spectral Counts Mean ± SD [a]				
Complement component C8 gamma chain	CO8G_HUMAN	P07360	17	±	7	13	±	1	1.33	0.6890
Complement component C9	CO9_HUMAN	P02748	55	±	5	42	±	2	1.31	**1.6499**
Complement factor B	CFAB_HUMAN	P00751	84	±	34	112	±	29	-1.34	**1.8012**
Complement factor H	CFAH_HUMAN	P08603	81	±	30	100	±	8	-1.24	**1.4773**
Complement factor H-related protein 1	FHR1_HUMAN	Q03591	29	±	3	29	±	2	1.01	0.0545
Complement factor H-related protein 2	FHR2_HUMAN	P36980	18	±	1	17	±	3	1.10	0.4122
Complement factor I	CFAI_HUMAN	P05156	43	±	7	39	±	12	1.09	0.4299
Corticosteroid-binding globulin	CBG_HUMAN	P08185	16	±	5	20	±	5	-1.26	0.6466
Fetuin-B	FETUB_HUMAN	Q9UGM5	5	±	3	5	±	1	-1.11	0.1413
Fibronectin	FINC_HUMAN	P02751	114	±	22	122	±	36	-1.07	0.5532
Ficolin-2	FCN2_HUMAN	Q15485	4	±	1	3	±	2	1.36	0.3381
Ficolin-3	FCN3_HUMAN	O75636	15	±	7	6	±	3	**2.52**	**1.2599**
Gelsolin	GELS_HUMAN	P06396	63	±	22	62	±	28	1.02	0.0845
Glutathione peroxidase 3	GPX3_HUMAN	P22352	5	±	2	5	±	3	-1.01	0.0070
Hemopexin	HEMO_HUMAN	P02790	206	±	80	198	±	26	1.04	0.4273
Heparin cofactor 2	HEP2_HUMAN	P05546	42	±	5	36	±	4	1.15	0.8660
Histidine-rich glycoprotein	HRG_HUMAN	P04196	42	±	15	39	±	10	1.07	0.2701
Hyaluronan-binding protein 2	HABP2_HUMAN	Q14520	5	±	1	3	±	1	1.41	0.4951
Insulin-like growth factor-binding protein complex acid labile subunit	ALS_HUMAN	P35858	17	±	1	16	±	1	1.06	0.3589
Inter-alpha-trypsin inhibitor heavy chain H1	ITIH1_HUMAN	P19827	163	±	9	145	±	23	1.13	**1.5433**
Inter-alpha-trypsin inhibitor heavy chain H2	ITIH2_HUMAN	P19823	213	±	21	208	±	31	1.02	0.3780
Inter-alpha-trypsin inhibitor heavy chain H3	ITIH3_HUMAN	Q06033	16	±	8	15	±	2	1.07	0.1726
Inter-alpha-trypsin inhibitor heavy chain H4	ITIH4_HUMAN	Q14624	155	±	24	141	±	37	1.10	0.9076
Kallistatin	KAIN_HUMAN	P29622	15	±	3	17	±	3	-1.19	0.5822
Kininogen-1	KNG1_HUMAN	P01042	51	±	11	52	±	10	-1.01	0.0767
Leucine-rich alpha-2-glycoprotein	A2GL_HUMAN	P02750	13	±	4	15	±	3	-1.22	0.5359

continued Table S2.										page 4/5
Protein Name	UniProt Entry Name	SwissProt-UniProt No.	EPIC-CRC case Pool			EPIC-Control Pool			FC[b]	VIP value[c]
			Spectral Counts Mean ± SD [a]			Spectral Counts Mean ± SD [a]				
Lumican	LUM_HUMAN	P51884	11	±	5	8	±	3	1.45	0.6035
Mannan-binding lectin serine protease 2	MASP2_HUMAN	O00187	4	±	1	2	±	1	**1.72**	0.5044
Mannose-binding protein C	MBL2_HUMAN	P11226	3	±	2	1	±	0	**3.30**	0.6181
Monocyte differentiation antigen CD14	CD14_HUMAN	P08571	3	±	2	3	±	1	-1.05	0.0553
N-acetylmuramoyl-L-alanine amidase	PGRP2_HUMAN	Q96PD5	15	±	1	9	±	4	**1.62**	**1.0321**
Pigment epithelium-derived factor	PEDF_HUMAN	P36955	28	±	5	36	±	9	-1.27	0.9778
Plasma kallikrein	KLKB1_HUMAN	P03952	30	±	2	30	±	2	-1.02	0.1722
Plasma protease C1 inhibitor	IC1_HUMAN	P05155	39	±	14	47	±	13	-1.21	0.8234
Plasma serine protease inhibitor	IPSP_HUMAN	P05154	12	±	2	14	±	7	-1.19	0.3736
Plasminogen	PLMN_HUMAN	P00747	78	±	26	94	±	23	-1.20	**1.1730**
Pregnancy zone protein	PZP_HUMAN	P20742	160	±	28	159	±	7	1.01	0.1208
Protein AMBP	AMBP_HUMAN	P02760	41	±	3	42	±	3	-1.04	0.3129
Protein Z-dependent protease inhibitor	ZPI_HUMAN	Q9UK55	3	±	2	3	±	2	1.09	0.0745
Proteoglycan 4	PRG4_HUMAN	Q92954	4	±	0	4	±	1	-1.07	0.0952
Prothrombin	THRB_HUMAN	P00734	59	±	11	62	±	12	-1.05	0.3455
Retinol-binding protein 4	RET4_HUMAN	P02753	121	±	114	80	±	20	**1.51**	**1.6648**
Serum amyloid A-4 protein	SAA4_HUMAN	P35542	27	±	8	34	±	0	-1.23	0.9243
Serum amyloid P-component	SAMP_HUMAN	P02743	27	±	4	31	±	6	-1.11	0.5130
Serum paraoxonase/arylesterase 1	PON1_HUMAN	P27169	56	±	6	64	±	14	-1.13	0.8397
Sex hormone-binding globulin	SHBG_HUMAN	P04278	7	±	4	7	±	0	1.11	0.1638
Tetranectin	TETN_HUMAN	P05452	6	±	2	7	±	2	-1.13	0.1823
Thyroxine-binding globulin	THBG_HUMAN	P05543	14	±	3	12	±	2	1.17	0.4485
Transthyretin	TTHY_HUMAN	P02766	241	±	43	229	±	75	1.05	0.5838
Vitamin D-binding protein	VTDB_HUMAN	P02774	154	±	16	164	±	6	-1.07	**1.0672**
Vitamin K-dependent protein C	PROC_HUMAN	P04070	4	±	3	4	±	0	-1.00	0.0018

continued Table S2.

Protein Name	UniProt Entry Name	SwissProt-UniProt No.	EPIC-CRC case Pool			EPIC-Control Pool			FC[b]	VIP value[c]
			Spectral Counts Mean ± SD [a]			Spectral Counts Mean ± SD [a]				
Vitamin K-dependent protein S	PROS_HUMAN	P07225	13	±	2	10	±	2	1.31	0.6861
Vitronectin	VTNC_HUMAN	P04004	43	±	7	42	±	7	1.03	0.1847
Zinc-alpha-2-glycoprotein	ZA2G_HUMAN	P25311	25	±	5	28	±	2	-1.12	0.5624

[a] Mean ± SD of three technical replicates

[b] FC, Fold Change, Spectral counts of CRC cases/controls; **bold**, FC≥1.5; ***bold-italic***, FC<-1.5

[c] VIP, Variable Importance in the Projection (PLS-DA analysis); **bold,** VIP>1

Table S3.

Relative amounts of proteins analysed by targeted proteomics (LC-SRM-MS) in the EPIC population not including Phase 2 samples (the number of subjects is given in parenthesis)

Candidate Biomarker	Relative amount [a] Mean ± SD		P-value [b]	Relative amount [a] Mean + SD		P-value [b]	Relative amount [a] Mean + SD		P-value [b]
	Controls	CRC Cases		Female Controls	Female CRC Cases		Male Controls	Male CRC Cases	
	(38)	(38)		(22)	(22)		(16)	(16)	
APOC2	8.13 ± 3.16	10.1 ± 7.27	0.26	8.14 ± 2.68	7.55 ± 3.47	0.50	8.07 ± 3.47	12.48 ±10.3	0.1
CLU	7.07 ± 1.85	7.57 ± 1.87	0.22	7.57 ± 1.59	7.28 ± 2.33	0.97	6.29 ± 1.91	7.81 ± 1.83	0.019
CO4-B	10.85 ± 6.08	11.89 ± 6.18	0.38	10.1 ± 4.47	10.52 ± 6.81	1	10.83 ± 8.07	11.56 ± 5.0	0.38
CO9	0.80 ± 0.34	0.91 ± 0.36	0.22	0.85 ± 0.3	0.87 ± 0.38	0.87	0.74 ± 0.38	0.86 ± 0.33	0.4
FETUA	2.6 ± 1.03	2.8 ± 0.89	0.26	2.72 ± 0.97	2.74 ± 1.04	0.76	2.79 ± 1.12	2.78 ± 0.9	0.89
MASP2	7.38 ± 2.35	7.69 ± 2.57	0.7	7.16 ± 1.93	6.99 ± 2.27	0.84	7.36 ± 2.59	7.91 ± 2.86	0.51
MBL2	1.13 ± 0.58	1.19 ± 0.52	0.56	1.2 ± 0.62	1.19 ± 0.57	0.97	1.07 ± 0.57	1.22 ± 0.54	0.53
PGRP2	0.54 ± 0.27	0.54 ± 0.16	0.38	0.59 ± 0.31	0.48 ± 0.18	0.22	0.48 ± 0.16	0.56 ± 0.14	0.088

[a] Analyte peak area/internal standard peak area

[b] Mann-Whitney U, two tailed

Table S4.

ROC analysis: AUC of various combinations of protein biomarkers in males [a]

Biomarker combination	AUC	95% CI	P-value	Sensitivity %	1-Specificity %
CLU + APOC2 + CO4-B	0.618	0.517 - 0.718	0.026	90	68
CLU + APOC2 + MASP2	0.622	0.521 – 0.723	0.021	88	68

[a] Only the combinations with P-value <0.05 are shown
