# Supplementary material for: Plasma clusterin as a candidate pre-diagnosis marker of colorectal cancer risk in the Florence cohort of the European Prospective Investigation into Cancer and Nutrition: a pilot study
Source: BMC Cancer. 2015 Feb 14;15:56. doi: 10.1186/s12885-015-1058-7 (PMC4334607; doi:10.1186/s12885-015-1058-7)
Supplement: Additional file 3: Figure S1. — PLS-DA analysis score scatter plot on proteins identified in the Phase 1 exploratory study after global proteome analysis. Figure S2. PLS-DA analysis score scatter plot on proteins identified in the Phase 2 EPIC study after global proteome analysis. Figure S3. Complement cascade: MetaCore “Network Enrichment analysis” on proteins with altered plasma levels (FC ≥ 1.5 or ≤ −1.5). Figure S4. SRM transition traces showing the separation of the selected peptides for the eight candidate biomarkers and the internal standard. Figure S5. Response linearity for the selected proteins after LC-SRM-MS analysis of increasing volumes of plasma. [file 12885_2015_1058_MOESM3_ESM.pdf]

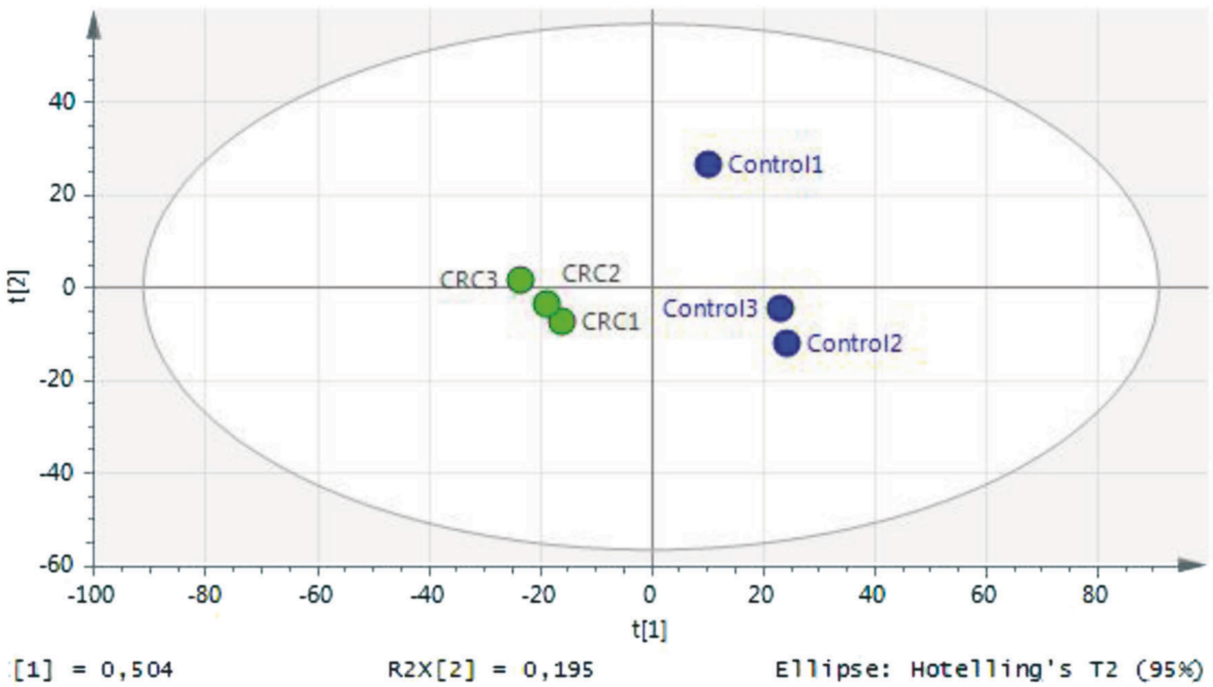

Figure S1

PLS-DA analysis score scatter plot on proteins identified in the Phase 1 exploratory study after global proteome analysis.

CRC, colorectal cancer

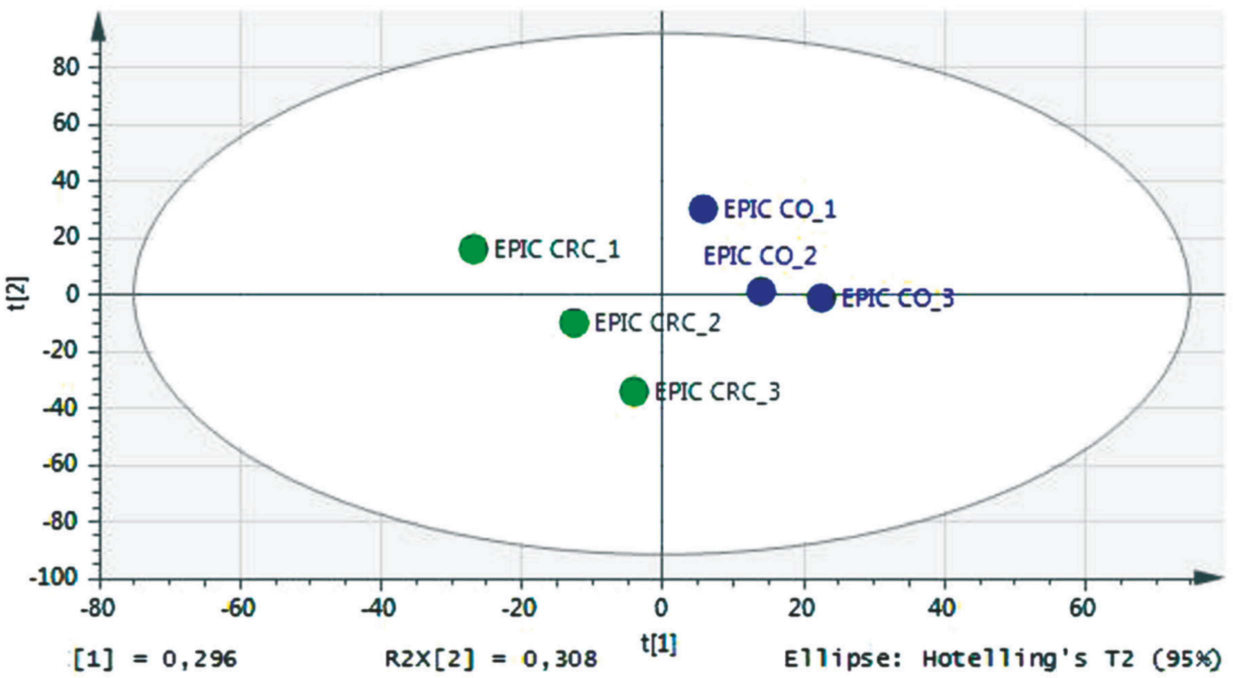

Figure S2

PLS-DA analysis score scatter plot on proteins identified in the Phase 2 EPIC study after global proteome analysis.

CRC, colorectal cancer
CO, controls

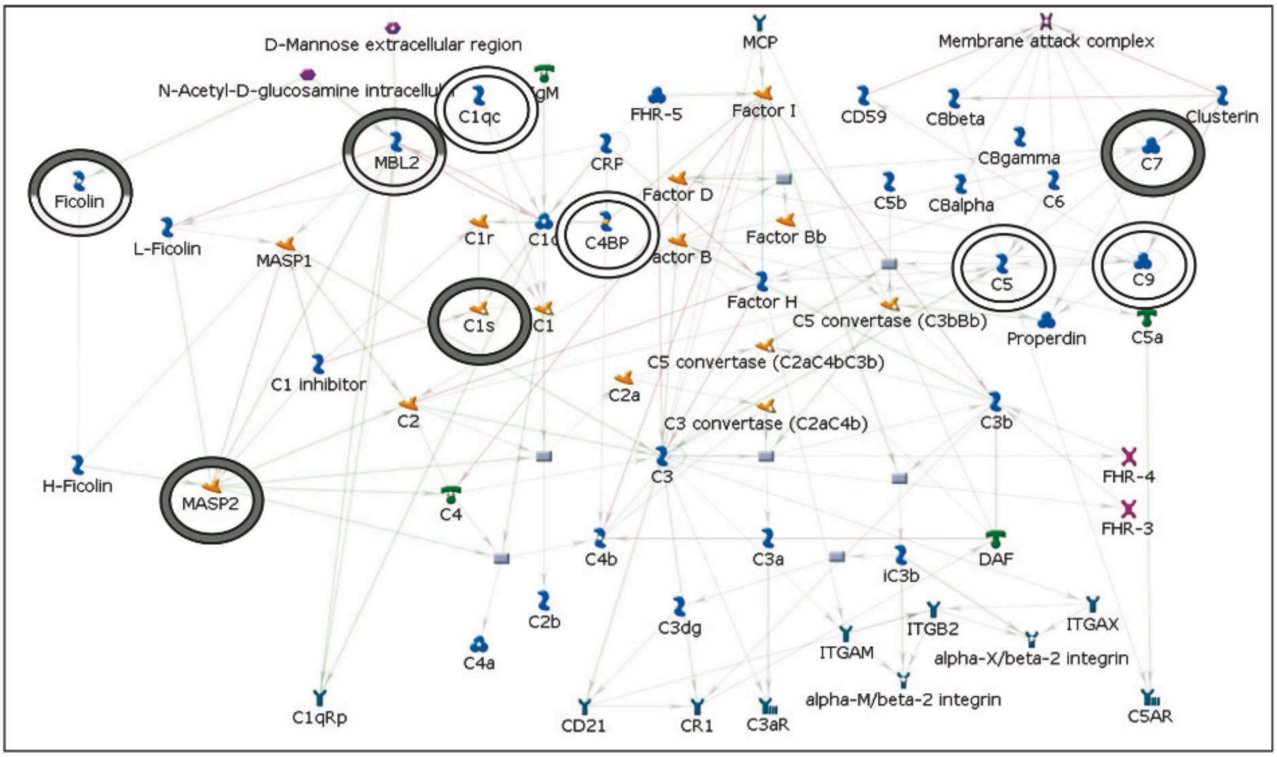

Figure S3

Complement cascade: MetaCore "Network Enrichment analysis" on proteins with altered plasma levels (FC ≥ 1.5 or ≤ -1.5). Individual proteins are represented as nodes of different shapes, according to their functional class. Lines between nodes indicate direct protein-protein interactions, the arrowheads indicating the direction of the interaction. Circled nodes denote proteins identified in this study (Phase 1 and Phase 2). White circles mark proteins identified only in the Phase 1 exploratory study; gray circles mark proteins identified only in the Phase 2 EPIC study; gray and white circles mark proteins identified both in the Phase 1 exploratory and Phase 2 EPIC studies.

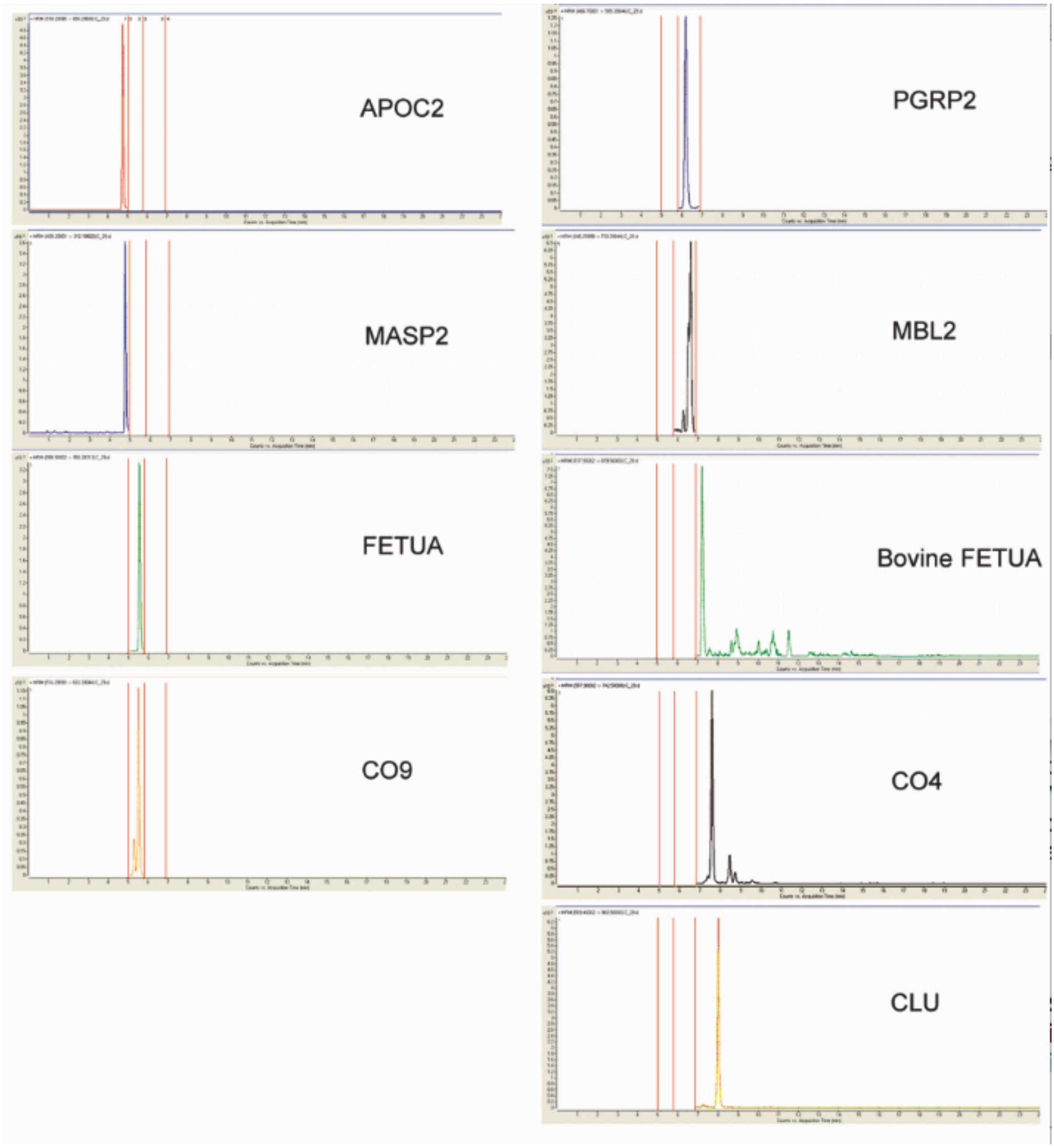

Figure S4

SRM transition traces showing the separation of the selected peptides for the eight candidate biomarkers and the internal standard bovine FETUA. Only the transitions used for relative quantitation are reported. Starting/ending points of the time segments are shown as red vertical lines.

APOC2, apolipoprotein C-II; MASP2, mannan-binding lectin serine protease 2; FETUA, alpha-2-HS-glycoprotein; CO9, complement component C9; PGRP2, N-acetylmuramoyl-L-alanine amidase; MBL2, mannose-binding protein C; Bovine FETUA, bovine alpha-2-HS-glycoprotein (internal standard); CO4, complement C4-B; CLU, clusterin.

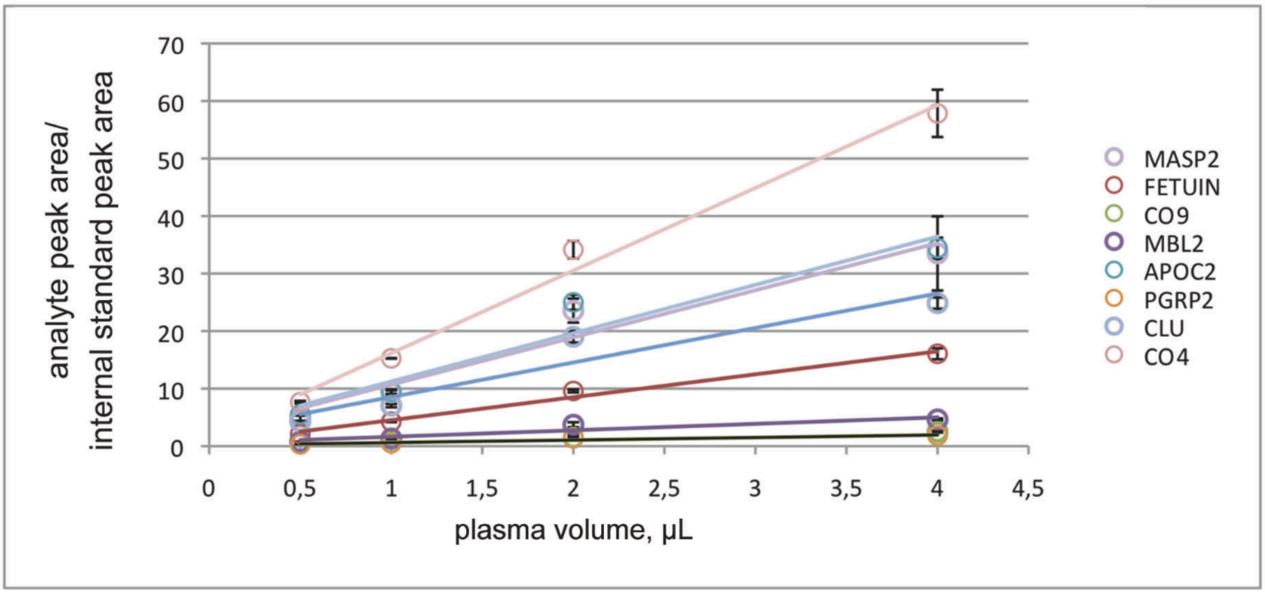

Figure S5

Response linearity for the selected proteins after LC-SRM-MS analysis of increasing volumes of plasma.

MASP2, mannan-binding lectin serine protease 2; FETUIN, alpha-2-HS-glycoprotein; CO9, complement component C9; MBL2, mannose-binding protein C; APOC2, apolipoprotein C-II; PGRP2, N-acetylmuramoyl-L-alanine amidase; CLU, clusterin; CO4, complement C4-B.
